# Role of defects in determining the magnetic ground state of ytterbium titanate

**DOI:** 10.1038/s41467-019-08598-z

**Published:** 2019-02-07

**Authors:** D. F. Bowman, E. Cemal, T. Lehner, A. R. Wildes, L. Mangin-Thro, G. J. Nilsen, M. J. Gutmann, D. J. Voneshen, D. Prabhakaran, A. T. Boothroyd, D. G. Porter, C. Castelnovo, K. Refson, J. P. Goff

**Affiliations:** 1 Department of Physics, Royal Holloway, University of London, Egham, TW20 0EX UK; 2 Institut Laue-Langevin, CS 20156, 38042 Grenoble Cedex 9, France; 3 ISIS Facility, Rutherford Appleton Laboratory, Chilton, Didcot, OX11 0QX UK; 4 Department of Physics, University of Oxford, Oxford, OX1 3PU UK; 5 Diamond Light Source, Harwell Science and Innovation Campus, Didcot, OX11 0DE UK; 6 Theory of Condensed Matter group, Cavendish Laboratory, University of Cambridge, Cambridge, CB3 0HE UK

## Abstract

Pyrochlore systems are ideally suited to the exploration of geometrical frustration in three dimensions, and their rich phenomenology encompasses topological order and fractional excitations. Classical spin ices provide the first context in which it is possible to control emergent magnetic monopoles, and anisotropic exchange leads to even richer behaviour associated with large quantum fluctuations. Whether the magnetic ground state of Yb_2_Ti_2_O_7_ is a quantum spin liquid or a ferromagnetic phase induced by a Higgs transition appears to be sample dependent. Here we have determined the role of structural defects on the magnetic ground state via the diffuse scattering of neutrons. We find that oxygen vacancies stabilise the spin liquid phase and the stuffing of Ti sites by Yb suppresses it. Samples in which the oxygen vacancies have been eliminated by annealing in oxygen exhibit a transition to a ferromagnetic phase, and this is the true magnetic ground state.

## Abstract (web summary)

Exploring the role of structural defect is essential to understand the exotic quantum spin phenoma in rare earth pyrochlores. Here the authors show oxygen vacancies can stabilise the spin liquid phase and reveal the ferromagnetic ground state when oxygen vacancies are eliminated in Yb_2_Ti_2_O_7_.

## Introduction

Yb_2_Ti_2_O_7_ has attracted intense experimental and theoretical interest as a model geometrically frustrated magnet with large quantum fluctuations^1–15^. The crystalline electric field gives a Kramers doublet ground state well separated from the excited states^4^, and symmetry considerations lead to an effective spin ½ Hamiltonian with four exchange constants^5^. In zero applied magnetic field there is a continuum of spin excitations as a result of quantum fluctuations^6,14,15^. In a magnetic field above *B* ~ 0.5 T well-defined spin waves are recovered, and it is possible to determine the exchange constants via inelastic neutron scattering^1^. It was originally proposed that there is no long-range magnetic order down to the lowest temperatures^7,8^, and that the dominant exchange is a ferromagnetic Ising-like term, with a large anisotropic term, leading to the intriguing proposal of a quantum spin ice^1^. Alternatively, it has been reported that the magnetic ground state is ferromagnetic^2,9–13^, and the values of the anisotropic g-tensor show that the moments are more easy-plane than easy-axis^16^.

The identification of different magnetic ground states for nominally stoichiometric samples clearly points towards the importance of low levels of structural disorder. This sample dependence is strongly reinforced in measurements of the heat capacity, where the existence of a sharp peak in the millikelvin range varies dramatically for different powder and single-crystal samples^11,17,18^. To date studies of structural disorder in Yb_2_Ti_2_O_7_ have focussed on the effect of stuffing Yb^3+^ ions on Ti^4+^ sites^19,20^. Recent studies have investigated the effect of pressure on the ground state of nominally stoichiometric and stuffed samples^21^. The fact that pressure appears to stabilise the ferromagnetic phase has been related to the chemical pressure associated with stuffing.

We have determined the defect structures in a range of oxygen-depleted, stuffed and nominally stoichiometric single crystals using diffuse neutron scattering, which is particularly sensitive to vacancies and displacements of oxygen ions. By correlating our results with magnetic diffuse scattering we are able to determine the effect of structural disorder on the spin correlations, and to unambiguously identify the true magnetic ground state of Yb_2_Ti_2_O_7_.

## Results

### Defect structures

All of our single-crystal X-ray diffraction data for stoichiometric Yb_2_Ti_2_O_7_, oxygen-depleted Yb_2_Ti_2_O_7−*δ*_ and stuffed Yb_2_(Ti_2−*x*_Yb_*x*_)O_7−*x*/2_ refine in the cubic pyrochlore structure, space group *Fd-3m*, see Table 1. The Yb and Ti ions are located on pyrochlore lattices, and there are two inequivalent O sites: O(1) located at the centre of the Yb tetrahedra and O(2) forming a corner-sharing lattice of octahedra surrounding Ti ions. The presence of high atomic number elements means that the sensitivity to the oxygen ions is limited, and it is not possible to distinguish between O(1) and O(2) vacancies. However, we note that oxygen depletion does not lead to an increase in lattice parameter, and this is the first difference between Yb_2_Ti_2_O_7−*δ*_ and the related oxygen-deficient pyrochlore Y_2_Ti_2_O_7−*δ*_ (ref. ^22^). In the case of Y_2_Ti_2_O_7−*δ*_, the increase in the lattice parameter was associated with the expansion of the Y tetrahedra around O(1) vacancies, and this is a clear indication that the defect structure of Yb_2_Ti_2_O_7−*δ*_ is different.

**Table 1:** Refinement of the average structures of stuffed Yb_2_(Ti_2−*x*_Yb_*x*_)O_7−*x*/2_, oxygen-depleted Yb_2_Ti_2_O_7−*δ*_, as-grown and oxygen-annealed Yb_2_Ti_2_O_7_ from the single-crystal X-ray structure factors measured at *T* ~ 300 K

	Stuffed	Depleted	As-grown	Annealed
Colour	Yellow/Brown	Black	Brown	Transparent
Space group	Fd-3m	Fd-3m	Fd-3m	Fd-3m
Lattice parameter	10.0838(14)	10.0216(16)	10.0271(2)	10.0313(17)
Yb1	1	1	1	1
Yb2	0.26(3)	0	0	0
Ti	0.74(3)	1	1	1
O(1)	1	1	1	1
O(2)	0.96	0.96(3)	1	1
*x*	0.3397(14)	0.3300(6)	0.3308(2)	0.3309(10)
*R*	4.01	1.92	4.23	2.60
*R*_W_	4.90	2.60	4.87	2.78

The fractional coordinates are given within the second origin choice of the Fd-3m space group: Yb1 (0.5,0.5,0.5), Yb2 (0,0,0), Ti (0,0,0), O(1) (0.375,0.375,0.375), O(2) (*x*,0.125,0.125). The goodness of fit is described by the crystallographic *R*-factor and the weighted *R*-factor, *R*_W_

**Fig. 1:**
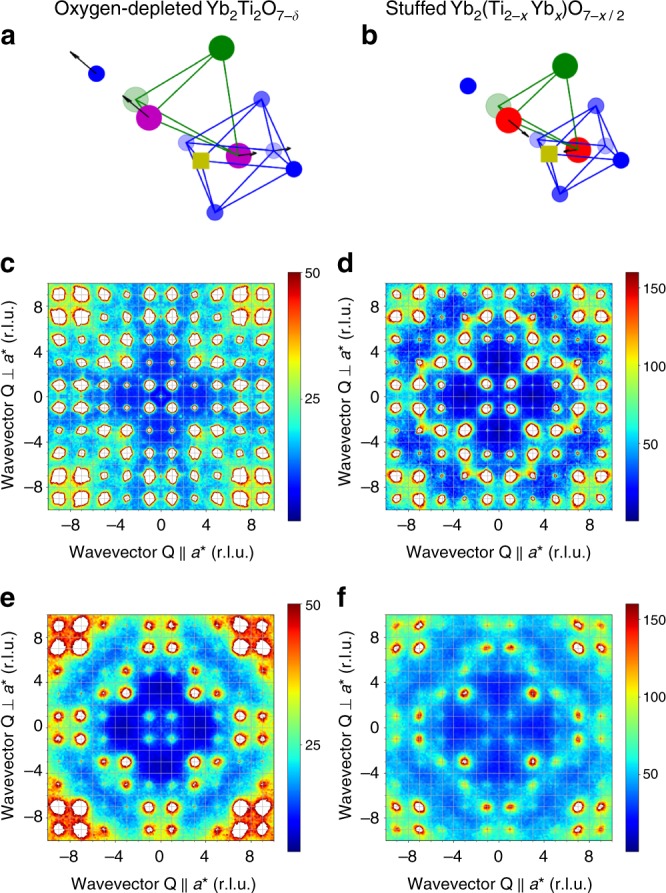
The structure of defects in Yb_2_Ti_2_O_7_. **a** For oxygen-depleted Yb_2_Ti_2_O_7−*δ*_ the oxygen vacancies (gold) are located on the O(2) sites (blue), and neighbouring Ti^4+^ (green) are replaced by charge compensating Ti^3+^ (purple) ions that are slightly displaced away from the vacancy. **b** For stuffed Yb_2_(Ti_2−*x*_Yb_*x*_)O_7−*x/2*_ neighbouring Ti^4+^ ions are replaced by Yb^3+^ (red) ions that are displaced towards the O(2) vacancy. The diffuse neutron scattering measured at *T* ~ 30 K in the (*hk*7) plane in **c** for oxygen depleted and **d** for stuffed samples is compared with the simulated scattering in **e** and **f**, calculated using the models shown in **a** and **b**, respectively. The contour diagrams are on a hot scale with an arbitrary maximum

The diffuse neutron scattering from oxygen-depleted Yb_2_Ti_2_O_7−*δ*_ is very sensitive to departures from ideal stoichiometry. Figure 1 presents the defect structure, diffuse scattering in the (*hk*7) plane, and the calculated scattering from Yb_2_Ti_2_O_7−*δ*_. The diffuse scattering pattern is very different to the comparable data set from Y_2_Ti_2_O_7−*δ*_, which was shown to arise from the defect cluster surrounding O(1) vacancies^22^. We are able to reproduce qualitatively the main features of the diffuse scattering using a Monte Carlo simulation, where isolated O(2) vacancies and charge compensating Ti^3+^ ions replace nearest-neighbour Ti^4+^ ions^23,24^. The presence of Ti^3+^ ions results in displacement of nearby O(2) ions such that the Ti^3+^–O(2) bond length, 2.03 Å, agrees with the values for Y_2_Ti_2_O_7−*δ*_ (ref. ^22^) and Ti_2_O_3_ (ref. ^25^). Thus, our single crystals grown from the melt using the optical floating zone technique in a reducing atmosphere have oxygen vacancies on the O(2) sites. Chemically, ytterbium exhibits polyvalency, existing in both Yb^3+^ and Yb^2+^ valence states, unlike yttrium which can exist only as Y^3+^. This is the origin of the dramatic difference in the nature of the defect structures. We note that a powder neutron diffraction study of Yb_2_Ti_2_O_7_ reduced at low temperature in a topotactic reaction with CaH_2_ also finds O(2) vacancies^26^.

Figure 1 also shows that the diffuse neutron scattering from stuffed Yb_2_(Ti_2−*x*_Yb_*x*_)O_7−*x*/2_ in the (*hk*7) plane is different. It is again possible to reproduce the scattering using a Monte Carlo simulation for O(2) vacancies, but with neighbouring Ti^4+^ ions replaced by Yb^3+^ ions, and with different displacements of surrounding ions. The Yb^3+^–O(2) bond length is larger than the Ti^3+^–O(2) bond length, as expected for the larger ionic radius of Yb^3+^. Recently, calculations for Yb_2_(Ti_2−*x*_Yb_*x*_)O_7−*x*/2_ using density functional theory have confirmed that O(2) vacancies are energetically stable relative to O(1) vacancies^27^.

**Fig. 2:**
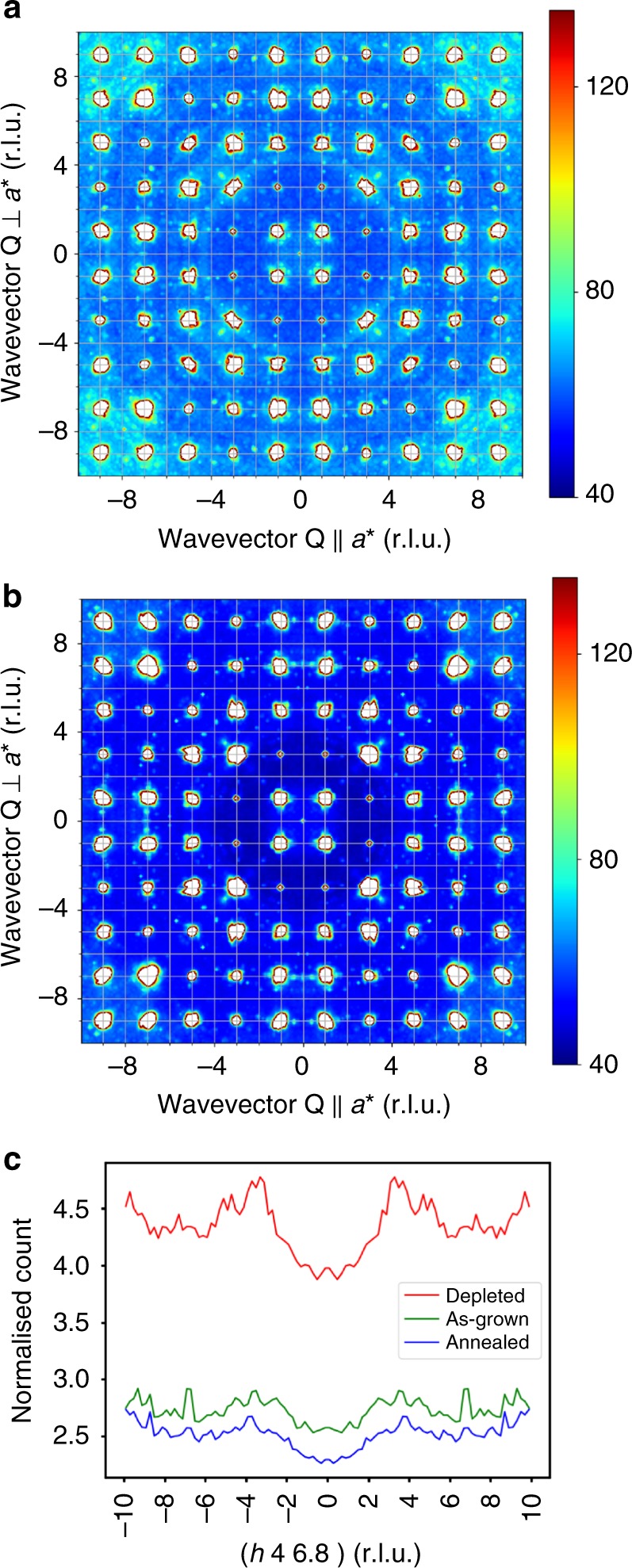
Structural diffuse scattering in nominally stoichiometric Yb_2_Ti_2_O_7_. **a** The diffuse neutron scattering from the as-grown, nominally stoichiometric sample measured at *T* ~ 30 K in the (*hk*7) plane contains weaker structural scattering resembling the scattering from the oxygen-deficient sample. **b** The same crystal measured after annealing in oxygen, showing that most of the diffuse scattering has been eliminated. The remaining scattering is attributed to inelastic scattering from phonons, see Supplementary Figure 1. **c** 1D cuts along [*h*,4,6.8] for the oxygen-depleted, as-grown and oxygen-annealed samples. This cut was chosen to minimise inelastic scattering. The broad structural diffuse features at *h* ~ ±4 are clearly present for the oxygen-depleted and as-grown samples, but are greatly reduced for the oxygen-annealed sample

The diffuse scattering from the nominally stoichiometric sample presented in Fig. 2a resembles the scattering from the oxygen-depleted sample, but is much weaker. Annealing in oxygen removes most of the intensity of the diffuse scattering, see Fig. 2b. The 1D cuts in Fig. 2c directly compare the diffuse scattering as a function of oxygen stoichiometry. We note that the diffuse scattering remaining after annealing in oxygen in the vicinity of the {337} and {777} reflections closely resembles the scattering observed for oxygen-annealed Y_2_Ti_2_O_7_ (ref. ^22^). We are able to demonstrate that this weak scattering arises from the inelastic scattering of phonons by examining the scattering observed in a single detector bank at a single orientation using the time-of-flight technique. Our first principles calculation of the phonon dispersion from Yb_2_Ti_2_O_7_ using the CASTEP code^28^, and the diffuse scattering arising from one-phonon excitations in our neutron single-crystal Laue diffraction using the approach described in ref. ^29^, is in excellent agreement with the arc of scattering emerging from the (337) reflection, see Supplementary Figure 1. This demonstrates that annealing in oxygen completely eliminates the structural component of the diffuse scattering for Yb_2_Ti_2_O_7_, and proves that the dominant defects in our nominally stoichiometric samples are O(2) vacancies.

### Magnetic ground state

**Fig. 3:**
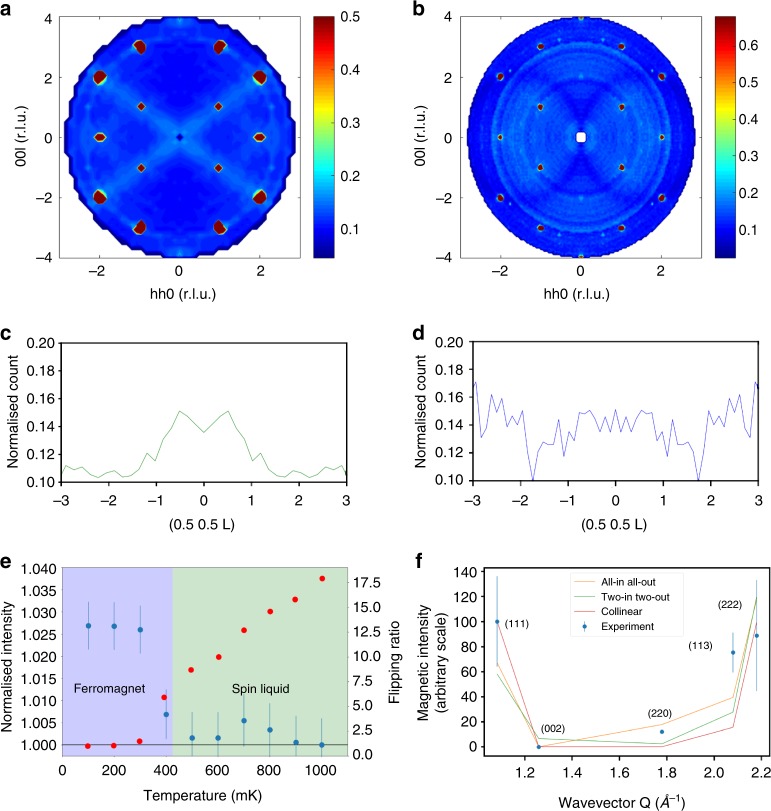
Spin correlations in nominally stoichiometric Yb_2_Ti_2_O_7_. **a** The unpolarised neutron scattering from the as-grown sample measured at *T* ~ 50 mK in the (*hhl*) plane, showing the characteristic rods of intensity along <111> directions. **b** Measurements under identical conditions from the same sample after annealing in oxygen shows the complete elimination of diffuse magnetic scattering. 1D cuts along [0.5,0.5,*l*] for **c** the as-grown and **d** the oxygen-annealed samples. The diffuse magnetic scattering from −1 < *L* < 1 observed for the as-grown sample completely disappeared for the oxygen-annealed sample. **a** and **b** are plotted with different scale bars because of different background levels, see **c** and **d**, from different volumes of copper in the beam. The dips in the scattering at *L* = ±1.8 in **d** result from the grazing-incidence/grazing-exit absorption by the copper plate, see Supplementary Figure 2c. **e** Temperature dependence of the integrated intensities of the (113) Bragg reflection normalised to unity at *T* ~ 1 K (blue), and the flipping ratio measurements (red), show that the oxygen-annealed sample enters a ferromagnetic phase below *T*_C _~ 425 mK. Error bars are the standard deviations derived using Poisson statistics. **f** Magnetic scattering intensities obtained by subtracting the integrated intensities at *T* ~ 1.2 K from those at *T* ~ 50 mK. The solid lines show the comparison with the data for the collinear ferromagnet^9^, the two-in two-out splayed ferromagnet^6^ and the all-in all-out splayed ferromagnet^10^. The magnetic intensity at (220) rules out the collinear ferromagnet, and the absence of magnetic intensity at (002) is inconsistent with the ice-like splayed ferromagnet

The magnetic diffuse scattering from the nominally stoichiometric sample in the (*hhl*) plane at *T* ~ 50 mK shown in Fig. 3a closely resembles the features reported previously in the spin liquid phase^2,30–33^. The same crystal was measured under identical conditions after annealing in oxygen, and the diffuse magnetic scattering completely disappeared (Fig. 3b). This can be seen more clearly in the corresponding 1D cuts through these data sets in Fig. 3c, d. The only variation in intensity away from Bragg reflections in the case of the oxygen-annealed sample can be explained by attenuation in the copper mounting plate, see Supplementary Figure 2 for further details. Polarisation analysis was employed throughout, and the flipping ratio was measured for the (111) Bragg reflection. For the oxygen-annealed sample the flipping ratio drops to unity below *T*_C_ ~ 425 mK, indicating a transition to a ferromagnetic phase, see Fig. 3e. In zero field, the formation of ferromagnetic domains below *T*_C_ results in depolarisation of the beam and, therefore, in Fig. 3 we show only the total scattering.

The temperature dependence of the integrated intensity at the (113) Bragg reflection normalised to its intensity at *T* ~ 1 K is also shown in Fig. 3e. There is a clear increase in intensity indicating the onset of long-range ferromagnetic ordering below *T*_C_ ~ 425 mK. We note that a single crystal of Yb_2_Ti_2_O_7_ cut from the same boule and annealed in oxygen under identical conditions exhibits a sharp peak in its heat capacity at *T*_C_ ~ 214 mK^13^. This is consistent with the purest samples in the literature^2,19^. The different *T*_C_ from neutron diffraction can readily be explained by the large hysteresis observed previously for this transition^6,9^ or possibly the accidental inclusion of low-energy fluctuations. The low-temperature magnetic intensities are consistent with the results of ref. ^10^ where the moments have a ferromagnetic component along <100>, but are splayed towards <111> directions so that the components perpendicular to the local easy axes are of the all-in all-out type. Other nominally stoichiometric samples have adopted different collinear^9^, nearly collinear^2^, and two-in two-out splayed^6,12^ ferromagnetic structures. The presence of strong magnetic intensity at (220) is inconsistent with the collinear ferromagnetic structure and, coupled with the absence of intensity at (002), this also rules out the two-in two-out ferromagnetic structure, see Fig. 3f.

### Spin correlations

**Fig. 4:**
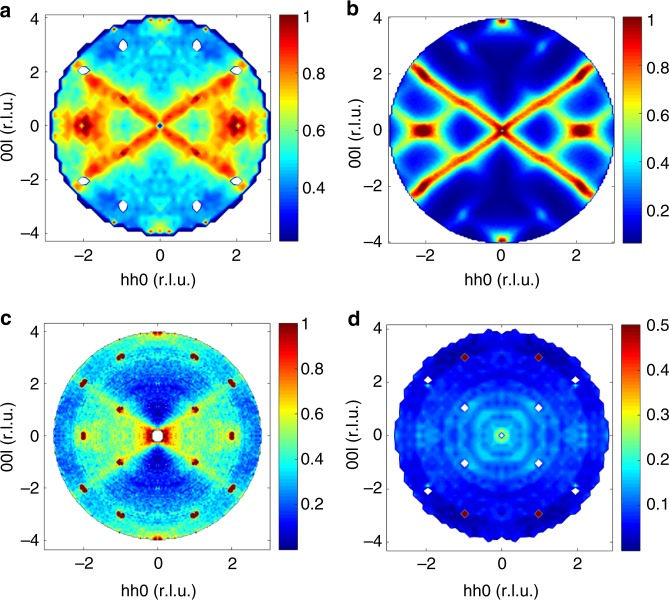
Spin-flip scattering as a function of composition. **a** The spin-flip scattering from the as-grown sample measured at *T* ~ 50 mK in the (*hhl*) plane exhibits the characteristic features associated with the spin liquid phase, including the rods along [111] and broad scattering near (220) and (004). **b** The scattering calculated using classical Monte Carlo at *T* ~ 450 mK and the exchange model of ref. ^30^. **c** The oxygen-depleted sample at *T* ~ 50 mK is similar to the scattering from the as-grown sample in **a**. **d** In contrast, these features are washed out for the stuffed sample at *T* ~ 50 mK, and instead the very broad diffuse scattering suggests mainly uncorrelated spins. The intensities are normalised to the size of crystal after background subtraction

For the as-grown sample there is no depolarisation of the beam at any temperature. We present the spin-flip scattering in the (*hhl*) plane at *T* ~ 50 mK in Fig. 4a. This is a particularly clean measurement of the spin correlations, since there is no structural scattering in this polarisation channel. Comparison with the diffuse scattering intensity calculated using classical Monte Carlo and the exchange constants from ref. ^30^ at *T* ~ 450 mK (above *T*_C_) shows excellent agreement with the data (Fig. 4b). In particular, the rods of diffuse scattering along the [111] directions and the broad features near (220) and (004) are reproduced well in the calculations. For this model below *T*_C_ the system orders in a ferromagnetic phase in agreement with previous MC simulations^30,34,35^. These exchange parameters place the system close to the boundary with the antiferromagnetic ψ_3_ phase, which has vanishing (002) scattering and strong (220) intensity, in agreement with our experiment. We note that this model is also consistent with the inelastic neutron scattering data from Yb_2_Ti_2_O_7_^13,30^.

For the oxygen-annealed sample above *T*_C_ the neutron beam remains polarised. The magnetic scattering in the spin-flip channel resembles the as-grown sample at a comparable temperature, see Supplementary Figure 3. In the case of the oxygen-depleted sample, there is no depolarisation of the beam down to base temperature, and Fig. 4c shows that the diffuse scattering in the (*hhl*) plane at *T* ~ 50 mK closely resembles the as-grown sample. Hence the presence of isolated O(2) vacancies appears to suppress the transition to the ferromagnetic phase and preserve the spin liquid behaviour down to the lowest temperature studied. The transition to the ferromagnetic phase is also suppressed for the stuffed sample. However, in this case the spin correlations are qualitatively different, see Fig. 4d. The rods of scattering along [111] directions are heavily suppressed, and the uniform diffuse scattering with a magnetic form factor instead points towards mainly uncorrelated spins. Hence the presence of additional magnetic Yb^3+^ ions on Ti^4+^ sites leads to additional exchange pathways and this suppresses the characteristic spin correlations associated with Yb_2_Ti_2_O_7_ in the spin liquid phase.

## Discussion

The observation of rods of scattering has been interpreted as a consequence of the proximity of competing antiferromagnetic ψ_2_ and ψ_3_ states^34,35^. It is interesting to observe that, at the classical Monte Carlo level, one of the most discriminating features between the different sets of exchange parameters proposed in the literature is the (220) scattering intensity, see Supplementary Figure 4. This is an antiferromagnetic feature that could be interpreted as either all-in all-out correlations in the transverse components of the incipient splayed ferromagnetic order, or as a competition between a collinear ferromagnet and the ψ_2_ or ψ_3_ antiferromagnetic phases. A comparison between quantum and classical numerical methods applied to Yb_2_Ti_2_O_7_ was presented in ref. ^34^, and the numerical linked cluster (NLC) expansion results differ from the classical Monte Carlo ones most strikingly by the change in (220) intensity. It would be interesting to determine the origin of the (220) intensity in NLC—whether it is quantum effects or the perturbative approximation favouring antiferromagnetic correlations—as it could become a smoking gun for quantum mechanical effects in Yb_2_Ti_2_O_7_.

The sensitivity of the magnetic ground state to modest hydrostatic pressures is also consistent with the proposed proximity to a phase boundary. It was recently reported that a nominally stoichiometric sample that did not order long range at ambient pressure, adopted a two-in two-out splayed ferromagnetic structure at *P* ~ 11 kbar^21^. It was suggested that the stabilisation of the ferromagnetic phase through the application of hydrostatic pressure implies that the observation of a ferromagnetic ground state arises when stuffing leads to chemical pressure. It is then surprising that the deliberately stuffed sample does not exhibit a ferromagnetic ground state at any pressure. We demonstrate that stuffing has the opposite effect of washing out the characteristic spin correlations in the spin liquid phase.

In summary, we have shown that low levels of intrinsic defects have a crucial role in determining the magnetic ground state of geometrically frustrated systems with enhanced quantum fluctuations. Single-crystal diffuse neutron scattering is an extremely sensitive probe of defect structures, and should be employed more widely in studies of model magnetic systems, particularly candidate quantum spin liquids. In the case of Yb_2_Ti_2_O_7_ we identify the introduction of isolated O(2) vacancies as a mechanism to stabilise the spin liquid, and annealing in oxygen as a means to obtain ultra-pure samples. Understanding the gapless continuum of quantum excitations at low temperature in the presence of competing classical ordered phases remains an outstanding challenge^15^. More generally, understanding defect structures in pyrochlores is becoming increasingly important since they can lead to spin glasses^36^, topological spin glasses^37^, and they may induce entirely novel quantum spin liquid phases^38–40^.

## Methods

### Sample preparation

A single crystal of Yb_2_Ti_2_O_7_ was grown at the Clarendon Laboratory by the floating zone technique^41^, and this as-grown nominally stoichiometric sample was dark brown. After studying the structure and magnetism, this crystal was annealed in O_2_ at a flow rate of 50 ml min^−1^ and a temperature of 1200 °C for 2 days, producing a transparent sample. Thermogravimetric analysis reveals a change in the oxygen stoichiometry of 0.38 ± 0.14%. The oxygen-depleted sample was grown and annealed in flowing mixed gas of hydrogen and argon, and this lead to a black crystal. The stuffed sample was grown by increasing the ratio of Yb_2_O_3_ to TiO_2_ in the starting materials, and in this case the crystal was yellow/brown.

### Structure determination

The average structures were determined by single-crystal X-ray diffraction using a Molybdenum source Oxford Diffraction diffractometer at Royal Holloway. A large CCD detector captures full reciprocal space coverage to a real space resolution of 0.6 Å. 3D profile analysis of each reflection is performed using the CrysAlisPro software^42^ and refinements of the average structure, including anisotropic thermal parameters, are performed using the Jana2006 software^43^. All refinements had R-factors less than 5%, exhibiting excellent fits to the data. Full details are available in the CIF files. The defect structures were studied by diffuse neutron scattering using the single-crystal diffractometer (SXD) at the ISIS pulsed neutron source at the Rutherford Appleton Laboratory. SXD combines the white-beam Laue technique with area detectors covering a solid angle of 2*π* steradians, allowing comprehensive diffraction and diffuse scattering data sets to be collected. Samples were mounted on aluminium pins and cooled to 30 K using a closed-cycle helium refrigerator in order to minimise the phonon contribution to the diffuse scattering. A typical data set required four orientations to be collected for 4 h per orientation. Data were corrected for incident flux using a null scattering V/Nb sphere. These data were then combined to a volume of reciprocal space and sliced to obtain single planar and linear cuts.

For the Monte Carlo code used to simulate the structural diffuse scattering, a crystal comprising 64 × 64 × 64 unit cells was generated from the average structure obtained from the refinement of the diffraction data. From a statistical perspective, the use of a large supercell helps us to average over the disorder in the system (self-average), and suppresses the background noise. O(2) ions are removed at random until we obtain the depletion concentration. Large displacements of neighbouring Ti^3+^ or Yb^3+^ ions are introduced by hand for the oxygen-depleted and stuffed samples, respectively. The distortion of the surrounding lattice is simulated using the balls and springs model in which hard spheres are connected to neighbouring ions by springs, and the simulation randomly displaces ions in order to minimise the elastic energy^23,24^. We were not able to reproduce the observed diffuse scattering using simulations with other point defects, such as O(1) vacancies^22^. We were further able to rule out antiphase domain boundaries^44^, since all of the pyrochlore Bragg reflections were sharp, and static disorder from the flexibility of the corner-sharing network^45^, since structural diffuse scattering is absent in our stoichiometric sample.

### Lattice dynamics

The phonon dispersion of Y_2_Ti_2_O_7_ was measured using the MERLIN spectrometer at the ISIS pulsed neutron source at the Rutherford Appleton Laboratory^46^. An 8 g single crystal was mounted on an aluminium plate with the (*hhl*) horizontal scattering plane and the sample was cooled to *T* ~ 30 K using a closed-cycle cryostat. MERLIN was set up using the G chopper running at 450 Hz, giving incident energies *E*_i_ ~ 120, 52, and 29 meV. The sample was rotated over 180° in 0.5° steps with data collected at each angle. Each data file was reduced using Mantid^47^ and files were combined using Horace^48^.

Calculations of the titanate phonon dispersion have previously been shown to be extremely challenging^49^. However, the zirconate pyrochlores avoid many of these issues^50^ so to produce the computational phonon dispersion of Yb_2_Ti_2_O_7_ and Y_2_Ti_2_O_7_ we have initially computed the dispersion for La_2_Zr_2_O_7_ (LZO) and mass substituted the La and Zr sites. The LZO dispersion was computed using density functional theory within the plane-wave pseudopotential approach as implemented in the CASTEP code^28^. The local density approximation was used with the default CASTEP ultrasoft pseudopotentials (version C9)^51^. The primitive electronic Brillouin zone was sampled with a Monkhorst-Pack grid^52^ of 4 by 4 by 4 points with a plane-wave cutoff of 600 eV. The lattice and atomic positions were relaxed such that the residual stresses and forces were less than 0.01 GPa and 0.005 eV/Å, respectively, with the quasi-Newton method^53^. The phonon dispersion was then computed using the finite-displacement, supercell method^54^ with a single cubic unit cell as the supercell. Mass substitution was performed as a post processing step using the CASTEP utilities. The time-of-flight one-phonon neutron scattering intensity was calculated for the particular sample orientation and scattering geometry on SXD^29^. Note that the inelastic scattering is calculated for energies above 1 meV in order to avoid singularities and, therefore, the calculations do not work precisely at Bragg reflections. For the heavily defective samples, the inelastic scattering is negligible in comparison to the structural diffuse scattering. However, for the pristine, oxygen-annealed sample, the inelastic scattering can be clearly identified.

### Magnetic scattering experiment

The magnetic diffuse scattering was measured using the D7 diffuse scattering spectrometer at the Institut Laue-Langevin in Grenoble. A pyrolytic graphite monochromator was employed to select cold neutrons of wavelength *λ* ~ 4.8 Å, and a beryllium filter was used to remove higher harmonics. Single crystals were mounted on a copper base plate inside a cryomagnet with the [00*l*] axis perpendicular to the plane of the plate, rotated so that the (*hhl*) crystallographic plane coincided with the horizontal scattering plane. A dilution refrigerator was used for temperature control. The instrument was operated in the diffraction configuration which integrates the dynamical response and, therefore, this technique measures the instantaneous correlations. Uniaxial polarisation analysis was employed in order to separate the magnetic and structural correlations. The polarisation direction was fixed normal to the scattering plane and this coincides with the [1–10] crystallographic direction. A polarising super-mirror (bender) and a Mezei flipper were inserted on the incoming neutron beam to select neutrons with a given spin. After the sample, the final polarisation was analysed using an array of polarising benders in front of the helium detectors. For each scattering vector, **Q**, the intensity was measured in both non-spin-flip (structural + magnetic correlations) and spin-flip (magnetic correlations only) channels. In the case of a ferromagnet in zero field, the formation of domains results in the depolarisation of the neutron beam, but in this case the non-spin-flip and spin-flip intensities can be combined to give the total (unpolarised) cross section. The integrated intensities of the Bragg reflections were obtained by summing the data above the background level in the vicinity of the reciprocal lattice points, and these data were normalised to data integrated in the same way well above *T*_C_.

### Magnetic scattering theory

In our work, we chose to focus on classical simulations of the spin system to compare to experiments. This choice was made for two reasons: (i) tuning the parameters in these simulations, one can obtain an excellent quantitative agreement with experiments; (ii) the ability to include quantum fluctuations is currently limited to the mean field random phase approximation and the perturbative NLC expansion techniques, where it may be unclear whether the differences with respect to the classical results are due to quantum effects or to the approximations involved. We performed classical Monte Carlo simulations of nearest-neighbour exchange Yb_2_Ti_2_O_7_ to compare with the experimental neutron scattering results. We neglected dipolar interactions as they are about 100 times weaker than the exchange coupling, and we are interested primarily in temperatures above the transition. We restricted our modelling to the crystal-field ground-state doublet, since neither temperatures nor neutron energies in the experiments are high enough to involve states above the doublet gap (~ 900 K^4,16^). The resulting model comprises classical Heisenberg spins *S* = ½ on the sites of a pyrochlore lattice, interacting via the generic exchange couplings *J*_1_, *J*_2_, *J*_3_, *J*_4_ allowed by the lattice symmetries^1,33^. Using single spin-flip updates, which are sufficient to ensure thermalisation above the critical temperature, and accounting for the single-ion anisotropy via an appropriate g-tensor, we computed the neutron structure factor of the system in the (*hhk*) plane. The SF and NSF components were computed with respect to the relevant neutron polarisation direction [1,−1,0]. We note that the numerical results can equivalently be interpreted as the thermodynamic average of instantaneous (*t* *=* 0) neutron scattering structure factor, or as the time-integrated (*ω* = 0) structure factor within the crystal-field ground-state doublet. We used the canonical 16-spin cubic unit cell, and system sizes *L* = 20 (main paper) and *L* = 16 (Supplementary Figure 4). At every temperature step, the autocorrelation function was allowed to drop twice to 0.01 to ensure equilibration before lowering the temperature further. Upon reaching the target temperature, an instance of the structure factor was computed, and the entire process was then repeated from high temperature (with a different random number seed) to generate statistically independent instances. All the figures in the paper were averaged over 1000 instances.

## Supplementary information

Supplementary Information

Peer Review File

## Data Availability

The X-ray data sets generated and analysed during the current study and the computer simulations are available from the corresponding author on reasonable request. The CIF files are available via Royal Holloway’s Figshare repository from 10.17637/rh.7499396 (oxygen annealed), 10.17637/rh.7499399 (as grown), 10.17637/rh.7499417 (oxygen depleted) and 10.17637/rh.7499435 (stuffed). All raw neutron data and the associated metadata obtained as a result of access to ISIS or ILL reside in the public domain, with ISIS or ILL acting as the custodian. The SXD data can be accessed from 10.5286/ISIS.E.49916341 and 10.5286/ISIS.E.58450172. The D7 data can be accessed from 10.5291/ILL-DATA.5-42-378 and 10.5291/ILL-DATA.5-42-419.

## References

[1] Ross KA (2011). Quantum excitations in quantum spin ice. Phys. Rev. X.

[2] Chang LJ (2012). Higgs transition from a magnetic Coulomb liquid to a ferromagnet in Yb_2_Ti_2_O_7_. Nat. Commun..

[3] Gingras MJP, McClarty PA (2014). Quantum spin ice: a search for gapless quantum spin liquids in pyrochlore magnets. Rep. Prog. Phys..

[4] Gaudet J (2015). Neutron spectroscopic study of crystalline electric field excitations in stoichiometric and lightly stuffed Yb_2_Ti_2_O_7_. Phys. Rev. B.

[5] Curnoe SH (2008). Structural distortion and the spin liquid state in Tb_2_Ti_2_O_7_. Phys. Rev. B.

[6] Gaudet J (2016). Gapless quantum excitations from an ice-like splayed ferromagnetic ground state in stoichiometric Yb_2_Ti_2_O_7_. Phys. Rev. B.

[7] Hodges JA (2002). First-order transition in the spin dynamics of geometrically frustrated Yb_2_Ti_2_O_7_. Phys. Rev. Lett..

[8] Gardner JS (2004). Spin-spin correlations in Yb_2_Ti_2_O_7_: a polarized neutron scattering study. Phys. Rev. B.

[9] Yasui Y (2003). Ferromagnetic transition of pyrochlore compound Yb_2_Ti_2_O_7_. J. Phys. Soc. Jpn..

[10] Yaouanc A (2016). A novel type of splayed ferromagnetic order observed in Yb_2_Ti_2_O_7_. J. Phys. Condens. Matter.

[11] Chang LJ (2014). Static magnetic moments revealed by muon spin relaxation and thermodynamic measurements in the quantum spin ice Yb_2_Ti_2_O_7_. Phys. Rev. B.

[12] Scheie A (2017). Reentrant phase diagram of Yb_2_Ti_2_O_7_ in a <111> magnetic field. Phys. Rev. Lett..

[13] Thompson JD (2017). Quasiparticle breakdown and spin Hamiltonian of the frustrated quantum pyrochlore Yb_2_Ti_2_O_7_ in a magnetic field. Phys. Rev. Lett..

[14] Peçanha-Antonio V (2017). Magnetic excitations in the ground state of Yb_2_Ti_2_O_7_. Phys. Rev. B.

[15] Chern, L. E., Kim, Y. B. Magnetic order with fractional excitations: applications to Yb_2_Ti_2_O_7_. Preprint at https://arxiv.org/abs/1806.01276 (2018).

[16] Hodges JA (2001). The crystal field and exchange interactions in Yb_2_Ti_2_O_7_. J. Phys. Condens. Matter.

[17] Yaouanc A (2011). Single-crystal versus polycrystalline samples of magnetically frustrated Yb_2_Ti_2_O_7_: specific heat results. Phys. Rev. B.

[18] D’Ortenzio RM (2013). Unconventional magnetic ground state in Yb_2_Ti_2_O_7_. Phys. Rev. B.

[19] Arpino KE (2017). Impact of stoichiometry of Yb_2_Ti_2_O_7_ on its physical properties. Phys. Rev. B.

[20] Ross K (2012). Lightly stuffed pyrochlore structure of single-crystalline Yb_2_Ti_2_O_7_ grown by the optical floating zone technique. Phys. Rev. B.

[21] Kermarrec E (2017). Ground state selection under pressure in the quantum pyrochlore magnet Yb_2_Ti_2_O_7_. Nat. Commun..

[22] Sala G (2014). Vacancy defects and monopole dynamics in oxygen-deficient pyrochlores. Nat. Mater..

[23] Welberry, T. R. *IUCr Monographs on Crystallography* (OUP, Oxford, 2004).

[24] Welberry TR (1985). Diffuse X-ray scattering and models of disorder. Rep. Prog. Phys..

[25] Abrahams SC (1963). Magnetic and crystal structure of titanium sesquioxide. Phys. Rev..

[26] Blundred GD, Bridges CA, Rosseinsky MJ (2004). New oxidation states and defect chemistry in the pyrochlore structure. Angew. Chem..

[27] Ghosh SS, Manousakis E (2018). Effects of stuffing on the atomic and electronic structure of the pyrochlore Yb_2_Ti_2_O_7_. Phys. Rev. B.

[28] Clark SJ (2005). First principles methods using CASTEP. Z. Krist..

[29] Gutmann MJ (2015). Computation of diffuse scattering arising from one-phonon excitations in a neutron time-of-flight single-crystal Laue diffraction experiment. J. Appl. Cryst..

[30] Robert J (2015). Spin dynamics in the presence of competing ferromagnetic and antiferromagnetic correlations in Yb_2_Ti_2_O_7_. Phys. Rev. B.

[31] Bonville P (2004). Transitions and spin dynamics at very low temperature in the pyrochlores Yb_2_Ti_2_O_7_ and Gd_2_Sn_2_O_7_. Hyperfine. Interact..

[32] Ross KA (2009). Two-dimensional Kagome correlations and field induced order in the ferromagnetic XY pyrochlore Yb_2_Ti_2_O_7_. Phys. Rev. Lett..

[33] Thompson JD (2011). Rods of neutron scattering intensity in Yb_2_Ti_2_O_7_: compelling evidence for significant anisotropic exchange in a magnetic pyrochlore oxide. Phys. Rev. Lett..

[34] Jaubert L (2015). Are multiphase competition and order by disorder the keys to understanding Yb_2_Ti_2_O_7_?. Phys. Rev. Lett..

[35] Yan H (2017). Theory of multiple-phase competition in pyrochlore magnets with anisotropic exchange with application to Yb_2_Ti_2_O_7_, Er_2_Ti_2_O_7_, and Er_2_Sn_2_O_7_. Phys. Rev. B.

[36] Andreanov A (2010). Spin glass transition in geometrically frustrated antiferromagnets with weak disorder. Phys. Rev. B.

[37] Sen A, Moessner R (2015). Topological spin glass in diluted spin ice. Phys. Rev. Lett..

[38] Savary L, Balents L (2017). Disorder-induced quantum spin liquid in spin ice pyrochlores. Phys. Rev. Lett..

[39] Wen JJ (2017). Disordered route to the Coulomb quantum spin liquid: random transverse fields on spin ice in Pr_2_Zr_2_O_7_. Phys. Rev. Lett..

[40] Martin N (2017). Disorder and quantum spin ice. Phys. Rev. X.

[41] Prabhakaran D, Boothroyd AT (2011). Crystal growth of spin-ice pyrochlores by the floating-zone method. J. Cryst. Growth.

[42] Agilent. *CrysAlis PRO* (Agilent Technologies Ltd, Yarnton, Oxfordshire, England, 2014

[43] Petrícek V, Dusek M, Palatinus L (2014). Crystallographic computing system JANA2006: general features. Z. Krist..

[44] Lau GC (2007). Structural disorder and properties of the stuffed pyrochlore Ho_2_TiO_5_. Phys. Rev. B.

[45] Trump BA (2018). Universal geometric frustration in pyrochlores. Nat. Commun..

[46] Bewley RI (2006). MERLIN, a new high count rate spectrometer at ISIS. Phys. B.

[47] Taylor, J. et al. Mantid, a high performance framework for reduction and analysis of neutron scattering data. *Bull. Am. Phys. Soc*. **57** (2012). 10.5286/software/mantid.

[48] Ewings RA (2016). HORACE: software for the analysis of data from single crystal spectroscopy experiments at time-of-flight neutron instruments. Nucl. Instrum. Methods Phys. Res. A.

[49] Rumint R (2016). First-principles calculation and experimental investigation of lattice dynamics in the rare-earth pyrochlores R_2_Ti_2_O_7_ (R = Tb,Dy,Ho). Phys. Rev. B.

[50] Lan G, Ouyang B, Song J (2015). The role of low-lying optical phonons in lattice thermal conductance of rare-earth pyrochlores: a first-principle study. Acta Mater..

[51] Lejaeghere K (2016). Reproducibility in density functional theory calculations of solids. Science.

[52] Monkhorst HJ, Pack JD (1976). Special points for Brillouin-zone integrations. Phys. Rev. B.

[53] Pfrommer BG (1997). Relaxation of crystals with the quasi-Newton method. J. Comput. Phys..

[54] Frank W, Elsasser C, Fahnle M (1995). *Ab initio* force-constant method for phonon dispersions in alkali metals. Phys. Rev. Lett..

