# Supplementary material for: Role of defects in determining the magnetic ground state of ytterbium titanate
Source: Nat Commun. 2019 Feb 7;10:637. doi: 10.1038/s41467-019-08598-z (PMC6367421; doi:10.1038/s41467-019-08598-z)
Supplement: Supplementary file 1 — Supplementary Information [file 41467_2019_8598_MOESM1_ESM.pdf]

Supplementary Information

Role of defects in determining the magnetic ground state of ytterbium titanate

D. F. Bowman[1], E. Cemal[1, 2], T. Lehner[1], A. R. Wildes[2], L. Mangin-Thro[2], G. J. Nilsen[2,3], M. J. Gutmann[3], D. J. Voneshen[3], D. Prabhakaran[4], A. T. Boothroyd[4], D. G. Porter[5], C. Castelnovo[6], K. Refson[1,3], J. P. Goff[1]*

[1]Department of Physics, Royal Holloway, University of London, Egham TW20 0EX, UK,
[2]Institut Laue-Langevin, CS 20156, 38042 Grenoble Cedex 9, France,
[3]ISIS Facility, Rutherford Appleton Laboratory, Chilton, Didcot OX11 0QX, UK,
[4]Department of Physics, University of Oxford, Oxford OX1 3PU, UK,
[5]Diamond Light Source, Harwell Science and Innovation Campus, Didcot OX11 0DE, UK,
[6]Theory of Condensed Matter group, Cavendish Laboratory, University of Cambridge, Cambridge CB3 0HE, UK.
*Corresponding author: Jon.Goff@rhul.ac.uk

This PDF file contains:

Supplementary Figures 1 to 4

Supplementary Table 1

Supplemenray References

Supplementary Figures

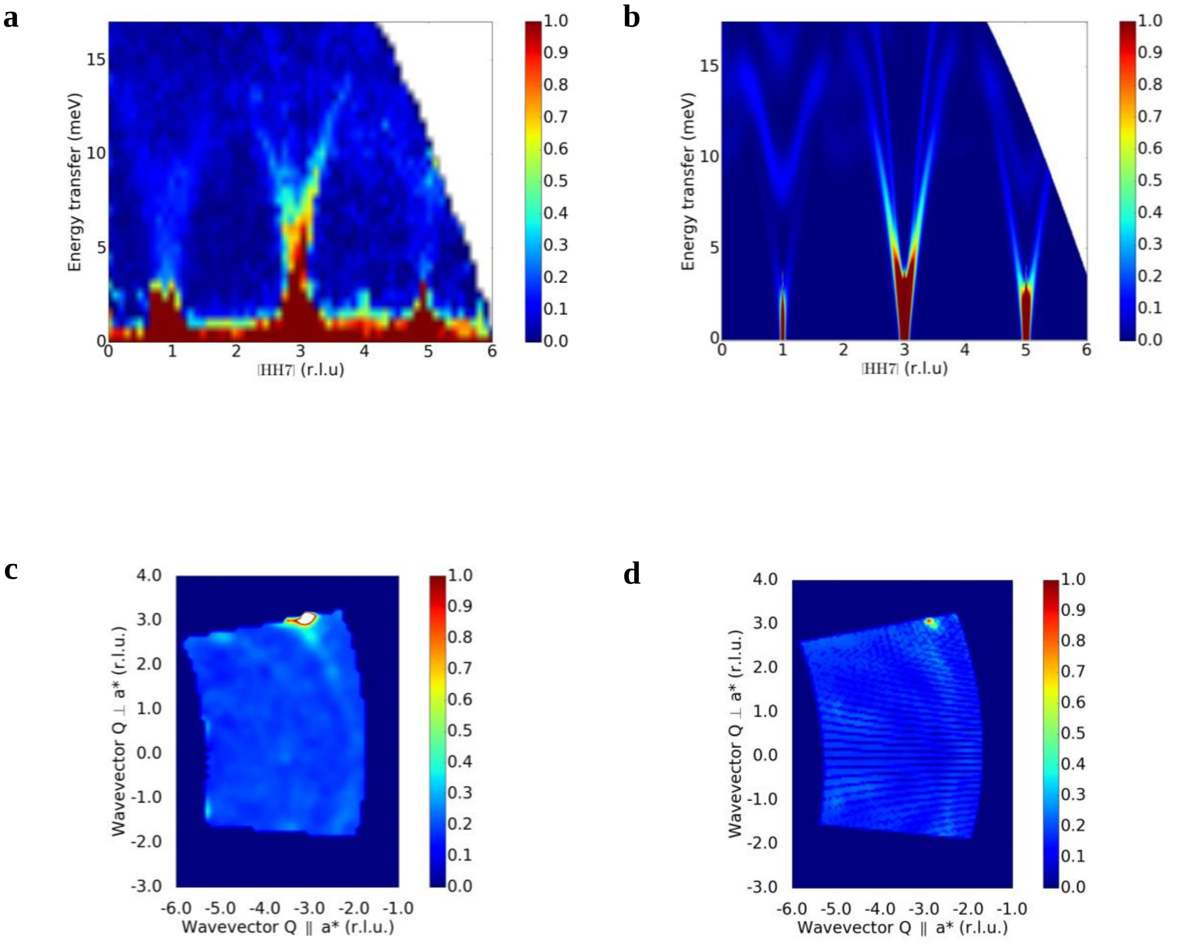

Supplementary Figure 1. The contribution to the diffuse scattering from phonons. (a) The phonon dispersion from $Y_2Ti_2O_7$ in the [*hh*7] direction measured at T ~ 30K using the MERLIN spectrometer at ISIS. (b) The neutron scattering intensity from $Y_2Ti_2O_7$ along the [*hh*7] direction calculated using the CASTEP code agrees well with the data, validating our first principles DFT lattice dynamics calculations. It is clear that the acoustic phonon intensity is particularly high near (337). (c) Inelastic neutron scattering intensity from $Yb_2Ti_2O_7$ in the vicinity of (337) observed in a single detector bank on SXD at T ~ 30K. The data have not been symmetrised. (d) Ab initio DFT simulations of the inelastic scattering predicted in this detector for this particular scattering geometry agrees well with the data, for example the arc of scattering emerging from the (337) reflection. Hence, it is possible to identify the remaining diffuse scattering from the oxygen-annealed $Yb_2Ti_2O_7$ sample as inelastic scattering from phonons.

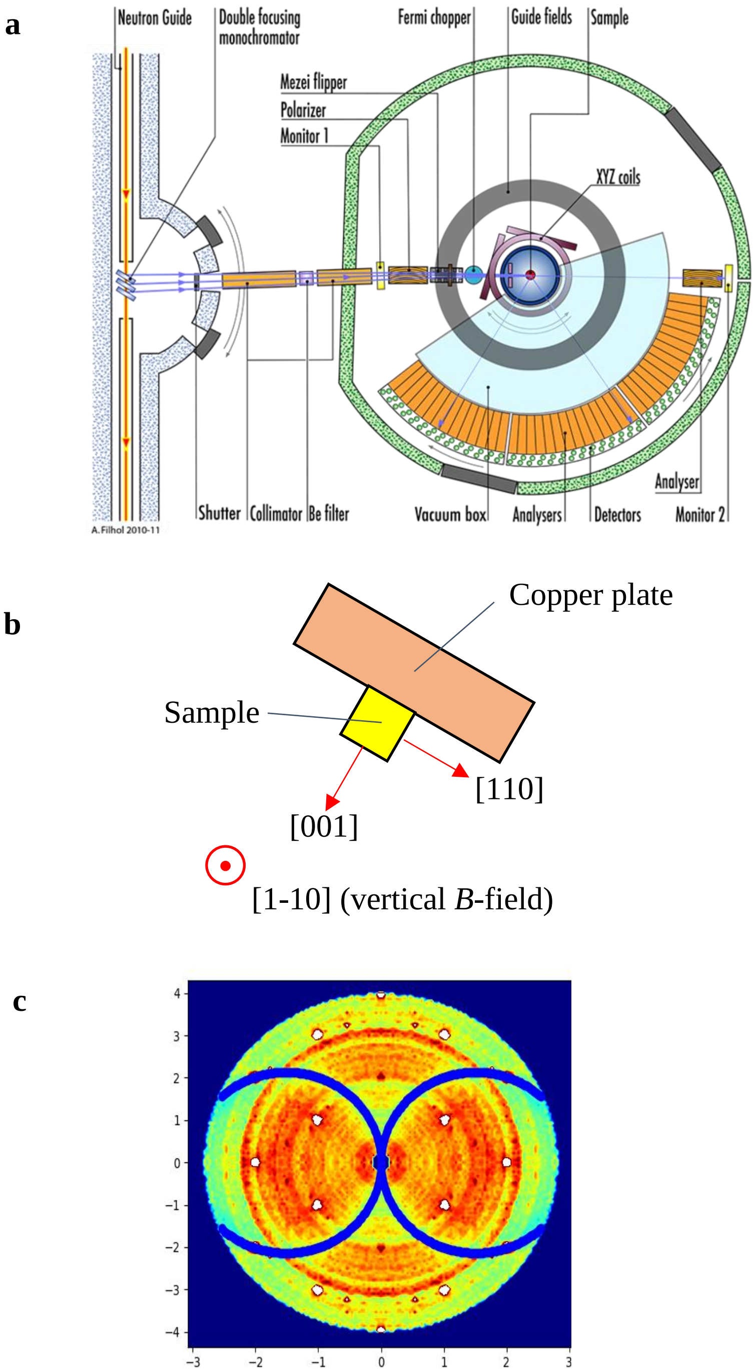

Supplementary Figure 2. The scattering geometry on D7[1]. (a) We employed uniaxial, vertical "Z-axis" polarisation analysis and in this case the XYZ coils were not used. (b) Sample orientation. Non-spin-flip scattering measures correlations of the structure and magnetic components parallel to [1-10], and the spin-flip scattering measures only correlations of the magnetic components perpendicular to [1-10]. Both measure a flat incoherent background. Hence the spin-flip scattering is particularly sensitive to any magnetic correlations. (c) The total scattering for the oxygen-annealed sample is essentially flat away from the Bragg reflections. The variation in intensity can be accounted for by attenuation in the copper plate, which is largest close to grazing incidence and grazing exit, shown as blue lines.

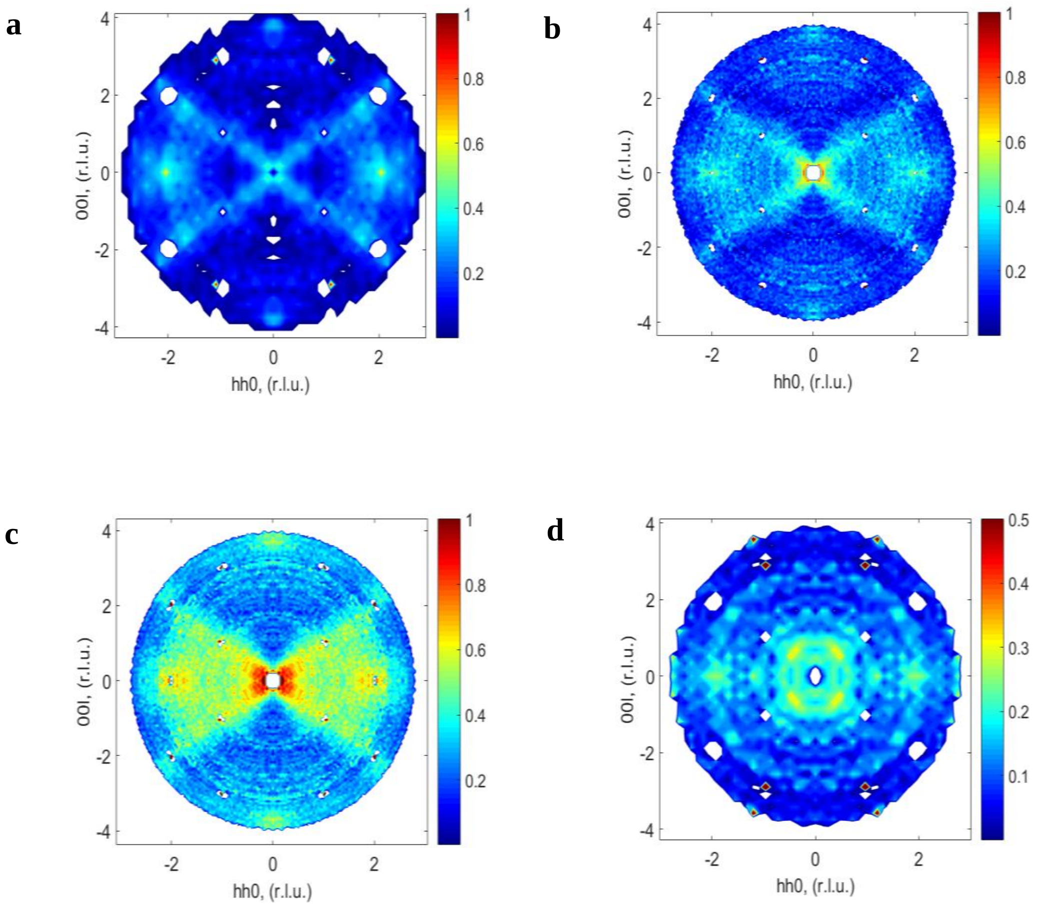

Supplementary Figure 3. Composition dependence of the spin-flip scattering at intermediate temperatures. (a) The magnetic diffuse scattering from the as-grown sample at T ~ 1.5 K is qualitatively similar to the pattern obtained at T ~ 50 mK in Fig. 4(a), but the intensity is weaker. (b) The magnetic diffuse scattering from the oxygen-annealed sample at T ~ 1.2 K is similar to (a), indicating that this sample enters the same spin liquid phase above T_C. (c) The magnetic diffuse scattering from the oxygen-depleted sample at T ~ 1.2 K again closely resembles (a), confirming that this sample resembles the as-grown sample at all temperatures. (d) The magnetic diffuse scattering from the stuffed sample at T ~ 1 K is featureless, indicating that the spin correlations are almost completely washed out. The intensities are normalised to the size of crystal after background subtraction.

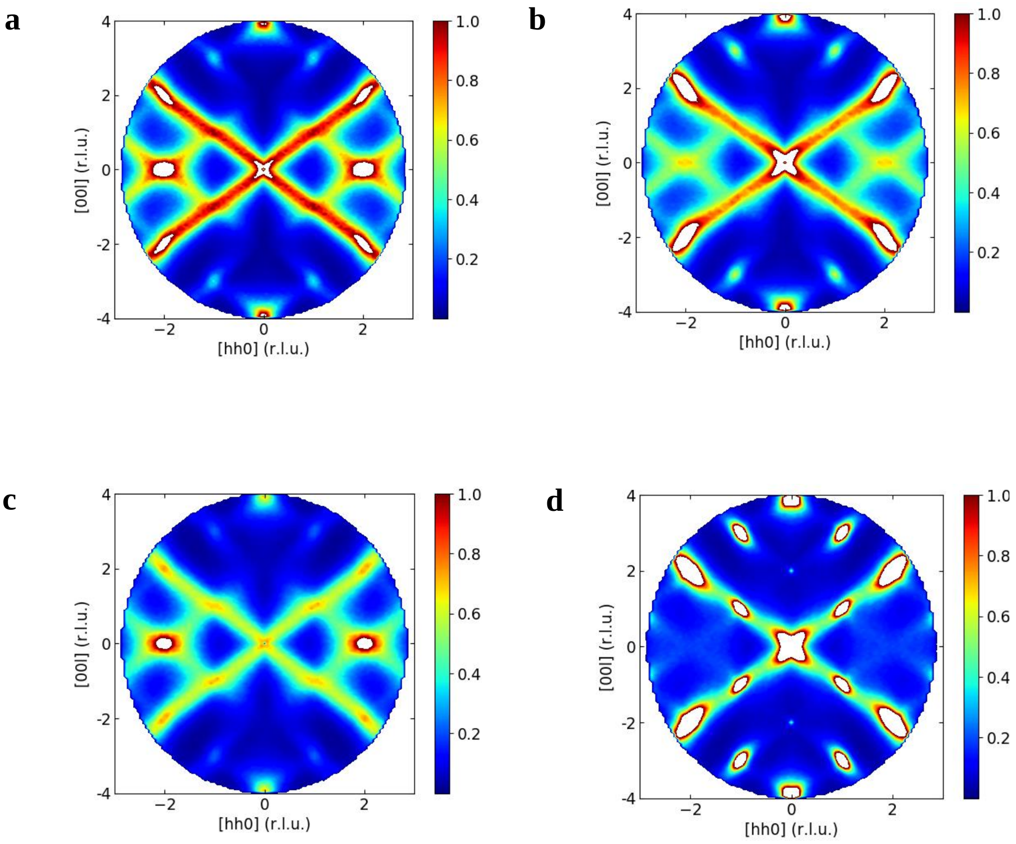

Supplementary Figure 4. Monte Carlo simulations of the SF scattering above T_C. The calculations were performed at T ~ 450 mK for the following models: (a) Ref. [2], (b) Ref. [3], (c) Ref. [4], and (d) Ref. [5]. At the level of these classical simulations, (a) and (c) are in excellent agreement with the data in Fig. 4(a), whereas the (220) intensity is remarkably weak for (b) and (d). A comparison between quantum and classical numerical methods applied to $Yb_2Ti_2O_7$ was presented in Ref. [4], and the NLC results differ from the classical Monte Carlo ones most strikingly by the reappearance of a strong (220) intensity for the choice of parameters in Ref. [5]. The difference with respect to the parameters in Ref. [2] is in the strength of the J_1 component for Ref. [5] and the J_4 component for Ref. [3]. It would be interesting to see NLC calculations carried out for the parameters in Ref. [3], to see if the intensity of the (220) scattering is once again enhanced.

Supplementary Table 1. Exchange constants and g-tensors for the models used in Supplementary Figure 4.

Model	J_1	J_2	J_3	J_4	$g_\perp$	$g_\parallel$
Ref. [2]	-0.03	-0.32	-0.28	0.02	4.09	2.06
Ref. [3]	-0.028	-0.326	-0.272	0.049	4.17	2.14
Ref. [4]	-0.0335	-0.22	-0.29	0	4.18	1.77
Ref. [5]	-0.09	-0.22	-0.29	0.01	4.3	1.79

Supplementary References

1. https://www.ill.eu/users/instruments/instruments-list/d7/description/instrument-layout/.
2. Robert, J. *et al*. Spin dynamics in the presence of competing ferromagnetic and antiferromagnetic correlations in $Yb_2Ti_2O_7$. *Phys. Rev. B* **92**, 064425 (2015).
3. Thompson, J. D. *et al*. Quasiparticle breakdown and spin Hamiltonian of the frustrated quantum pyrochlore $Yb_2Ti_2O_7$ in a magnetic field. *Phys. Rev. Lett.* **119**, 057203 (2017).
4. Jaubert. L. *et al*. Are Multiphase Competition and Order by Disorder the Keys to Understanding $Yb_2Ti_2O_7$? *Phys. Rev. Lett.* **115**, 267208 (2015).
5. Ross, K. A. *et al*. Quantum excitations in quantum spin ice. *Phys. Rev. X* **1**, 021002 (2011).
