# Supplementary material for: Role of defects in determining the magnetic ground state of ytterbium titanate
Source: Nat Commun. 2019 Feb 7;10:637. doi: 10.1038/s41467-019-08598-z (PMC6367421; doi:10.1038/s41467-019-08598-z)
Supplement: Supplementary file 2 — Peer Review File [file 41467_2019_8598_MOESM2_ESM.pdf]

Journal peer review information: *Nature Communications* thanks Tyrel McQueen, and the other anonymous reviewer(s) for their contribution to the peer review of this work. [Peer reviewer reports are available.]

Reviewers' comments:

Reviewer #1 (Remarks to the Author):

The paper reports the defect structures in oxygen depleted and Yb stuffed samples of Yb2Ti2O7 by means of neutron diffuse scattering investigation. The characteristics of the diffuse scattering are compared with the Monte Carlo calculation results to reveal the structural distortions in both cases are associated with isolated O(2) vacancies replacing the nearest-neighbour Ti4+ ions either with the charge compensating Ti3+ or with the Yb3+, respectively.
The paper then reports the diffuse scattering observed in as-grown (nominally stoichiometric) sample qualitatively resembles that of the oxygen depleted sample. The most of the diffuse scattering, except the phonon diffuse scattering, is removed when the sample was annealed in oxygen. They then conclude that the dominant defects in the nominally stoichiometric samples are O(2) vacancies.
To correlate these defect structures with the magnetic ground state and correlations, unpolarized and polarized neutron magnetic scattering investigations on these samples have been performed. While at 50 mK the magnetic diffuse scattering from the as-grown sample resembles that of the previously reported spin liquid phase with the rods of scattering along [111] directions, in the annealed sample the diffuse scattering disappeared and ferromagnetic ground state below Tc ~ 425mK is realized. However above Tc the annealed sample also shows diffuse magnetic scattering resembling that of the as-grown sample at a comparable temperature. The oxygen depleted sample does not order down to 50 mK and the diffuse magnetic scattering at this base temperature closely resembles that of the as-grown sample.
In the stuffed sample the transition to the ferromagnetic phase is also suppressed, but in contrast to the oxygen depleted sample it only shows an uniform magnetic diffuse scattering pointing to mainly uncorrelated spins.
In summary the paper highlights the important role of low level intrinsic defects in determining the magnetic ground state of the pyrochlore systems being a model geometrically frustrated systems with quantum fluctuations. The introduction of isolated O(2) vacancies are identified as a mechanism to suppress the ferromagnetic ordered ground state, which is realized in the ultra-pure sample of Yb2Ti2O7 obtained after annealing in oxygen.
All the experimental results are carefully compared with the theoretical calculations and thoroughly discussed.
The findings presented in the paper provides a credible scenario in understanding the long standing controversies on the different ground states reported in nominally stoichiometric Yb2Ti2O7 samples, hence it can be regarded as a very important contribution to the investigation of pyrochlore compounds as model systems for geometrical frustration in three dimensions. The paper thus definitely warrant publication in Nature Communications, after the authors have considered following points.

1) The presentation of the results in contour diagrams is appropriate to show the global character of the diffuse scattering, but makes the quantitative comparison between the different samples difficult, when the color scales are not given explicitly.
For example
the comparison of Fig.1 (c) and Fig. 2 (a) as described in the main text (on page 2, line 5 from the bottom) ' …, but much weaker.' or
the comparison of Fig. 3 (a) and (b) as described in the main text (on page 3, line 12 from the top) ' …and the diffuse magnetic scattering completely disappeared.'
can not be easily endorsed by just looking at the different contour plots.
A comparison in the form of characteristic cuts using the same scale, as has been employed in the Fig 2 (a) of Ref. 19, could clarify the quantitative differences of the sample dependent diffuse scattering as mentioned in the main text.

In addition the information on the size of the samples used for the neutron scattering experiments on Yb2Ti2O4 would be desirable to judge the intensity comparison of the different samples.

For example comparing the contour diagrams of Fig. 4 (a), (c) and (d), the overall spin-flip intensities of the stuffed sample (d) seem to be much higher than in other two samples, if the common color scale is assumed for these diagrams. Does this indicate a larger magnetic moment in the stuffed sample or is it due to the different size of the samples?

Providing an overall scaling of the observed intensities, e.g. on a nuclear Bragg peak, would certainly solve all the problems and would add essential information on the intensity levels of the diffuse structural and magnetic scattering discussed in this paper.

2) Additional refinement results for the as-grown sample in Table 1 may provide important information on the level of O(2) vacancies in �even so called’ nominally stoichiometric $Yb_2Ti_2O_7$ samples.

3) Are the error bars for Fig.3 (c) smaller than the data points?
Likewise, are the error bars in Fig 3 (d) for the (002) and (220) intensities smaller then the data points?

4) Typo:
on page 2, line 19 from the top, ‘ ….. presented in Fig. 1(b) ….’ should read ‘ ….. presented in Fig. 1(c) ….’ .

Reviewer #2 (Remarks to the Author):

This manuscript describes a study of the influence of structural and chemical disorder on the spin correlations of $Yb_2Ti_2O_7$. $Yb_2Ti_2O_7$ is a material which has attracted a lot of interest over an extended period due to its magnetic frustration and possible link to quantum spin liquid phases. Sample dependence has been a significant issue in studies of $Yb_2Ti_2O_7$ and the role of disorder in determining its magnetic properties is not well understood.

This work adds new evidence to the discussion of disorder in $Yb_2Ti_2O_7$ by presenting measurements of both the structural and magnetic diffuse scattering, for various $Yb_2Ti_2O_7$ samples and showing the relationship between the two. In particular, it is shown that two different types of disorder (oxygen deficiency and Yb stuffing) have different effects. Both types of disorder suppress the ferromagnetic ordered ground state but while oxygen deficiency promotes a correlated spin liquid state, Yb stuffing suppresses correlations altogether. The authors' present evidence that the true ground state of stoichiometric $Yb_2Ti_2O_7$ is ferromagnetically ordered.

This work provides a lot of new evidence about the role of disorder in $Yb_2Ti_2O_7$ and its magnetic ground state and it will be of considerable interest to the community studying this material. Since $Yb_2Ti_2O_7$ has become something of an important test case for frustrated quantum magnets in 3 dimensions, these results will also be of broader interest. More generally, the interplay of frustration, quantum fluctuations and quenched disorder is an increasingly studied topic at present and this work is of interest from that point of view. I therefore think that this work is of sufficient novelty and importance to merit publication in Nature Communications, if the authors can satisfactorily address the points I have listed below.

There are a few things which I think the authors need to address. These are:
1) The authors estimate the transition temperature for their oxygen annealed sample at 425mK. This is much higher than previous estimates of Tc for $Yb_2Ti_2O_7$ which generally fall between 200-300mK. Most of the published estimates of Tc are based on a sharp peak in the heat capacity whereas the present paper uses the onset of a magnetic Bragg peak. It is therefore important to know whether the

difference in estimates of Tc is due to the different method of estimation or whether the authors' sample really has a much higher Tc than everyone else's. This would be clarified if the authors could show some heat capacity measurements for their samples for comparison with the existing literature.

2) The authors' neutron scattering refinement finds a splayed ferromagnetic order with the splaying being of an "all in all out" (AIAO) rather than "two in two out" (2I2O) form. They state that this AIAO splayed FM order is reproduced in their classical Monte Carlo simulations. This surprises me. The classical phase diagram of the pyrochlore nearest neighbor anisotropic exchange model has been studied in some detail in previous works (particularly Ref. 31 of the manuscript) with only one splayed FM phase found, and this phase has 2I2O splaying. In fact, 2I2O rather than AIAO splaying is expected on quite general symmetry grounds. I also note that Ref. 26 which did Monte Carlo simulations using the same parameter set as the present work seems to find 2I2O splaying (see Figs. 7 c and d of Ref. 26). Given all this, it is quite a striking result if the authors have an FM phase with AIAO splaying in their classical simulations. They should present further evidence to support this and, ideally, an explanation of how such a state can be stabilised. For supporting evidence, I think it would be useful if they showed a plot from their simulations of the sum of the spin projections on their local <111> axes (spin ice axes) which should vanish in a 2I2O splayed state but be finite in an AIAO splayed state.

3) When the authors write in the introduction "...the latest values of the g-tensor show that the moments are more easy plane than easy axis", I think this slightly misrepresents the discussion about Yb2Ti2O7 in the literature. It has been known for a long time that the g-tensor is predominantly easy plane (see e.g. Hodges et al, JPCM 13, 9301 (2001)). While estimates of the g-tensor parameters have varied a bit, every study of which I am aware has found g_{xy}>g_z. The point of significant disagreement has not been the g-tensor but the relative strength of different exchange interactions with Ref. 1 of the manuscript finding quite a strong Ising term, but Refs. 13 and 26 finding that the Ising term is rather weak. I think the introduction should be modified to better reflect the discussion in the literature.

4) The abstract describes Yb2Ti2O7 as the "quantum analogue" of a classical spin ice. I am not sure this statement is justified. Although connections have been made between Yb2Ti2O7 and spin ice in the past, if recent estimates of the exchange parameters are correct (e.g. Ref. 13 of the manuscript) then the spin-ice-like Ising exchange in Yb2Ti2O7 is quite weak, which would suggest that the physics of Yb2Ti2O7 has little to do with spin ice, apart from being on the same lattice.

Since this paper provides new and interesting insights into a topic of quite broad interest, I will be happy to recommend it for publication in Nature Communications if the authors can satisfactorily address the points above.

Reviewer #3 (Remarks to the Author):

The rare earth pyrochlore magnets are benchmark materials for exploring the physics of geometrically frustrated lattices, and the interplay between single ion and emergent collective magnetic states. Recently, much work on compounds including Nd2Zr2O7, Pr2Zr2O7, and Yb2Ti2O7 (in journals from Nat. Commun. to PRX and PRL and even Acta Materialia) has shown that seemingly small variations in synthetic procedure and composition have an outsize impact on the resultant physics. As such, this is a timely contribution.

This manuscript's major claims are:

1) that defects play a pivotal role in determining the magnetic ground state of the putative quantum spin ice Yb2Ti2O7,
2) that the ferromagnetic state is the "true" ground state,

3) that oxygen vacancies can exist and favor the formation of a spin liquid (SL) state,
4) and that Yb stuffing suppresses the SL state.

Claim 1 has been known for many years, particularly with respect to the divergence (or not) in specific heat at T ~ 0.27 K, and claim 2 appears to be the community consensus. It is on the third and fourth claims that this manuscript aims to move the field forward -- prior work on Yb2Ti2O7 has found numerous types of defect structures, but the direct correlation between these and the physics has remained elusive. Part of the challenge is simply the sheer number of observed defect structures -- not just point defects (e.g. site mixing, oxygen vacancies) but also more recently a range of extended defects (antiphase domain boundaries observed in STEM, cooperative tetrahedral motion observed in electron diffraction).

Diffuse scattering is an excellent tool to probe both point and extended defect structures, and the data in this manuscript appears comprehensive. Further, neutron scattering (particularly the spin-flip variety) is a superb tool by which to probe the magnetic state of Yb2Ti2O7. Thus in principle the *data* of this manuscript provide the pieces needed resolve a number of long-standing questions about this material.

However, as it stands, the present level of analysis of the data, as well as a couple of defining measurements and references to the literature, are missing, and significantly weaken this manuscript. Specific technical points in no particular order:

1) For the structures reported in Table 1 from X-ray data: is this from powder or single crystal data? in either case, significant information about how the data was collected, reduced, and analyzed, is missing. If it is single crystal data, how the integration and scaling was done, how absorption corrections were applied, and how symmeterization was done is all missing; as are the unweighted R and chi^2 metrics, etc. Whether powder of single crystal, the refinement also includes atomic displacement parameters which are missing and not reported (and correlate with the sought after occupancies!), missing uncertainties in the O(2) occupancies, and missing atomic coordinates for each crystallographically distinct atomic position (including the critical refinable oxygen position).

2) Also, this is a cubic material so a single measurement of the lattice parameter 100% correlates with various common diffractometer alignment errors -- how was this accounted for in mesauring the cubic parameters? What was actually used for the X-ray measurements "The average structures were determined using x-ray diffraction at Royal Holloway" is not an adequate description of what was used and how the measurements were carried out.

3) The agreement between the models and the data appear reasonably good in Fig. 1. Crucially, however, this does not mean that other physically plausible models can be ruled out. More specifically, it appears a majority of the diffuse scattering appears to arise due to correlated movement of metal ions. This might be due to a point defect (e.g. an oxygen vacancy or a Yb-for-Ti stuffing, as claimed), but could it not also arise around the other types of defects observed in the literature -- e.g. antiphase domain boundaries (where definition of A and B sites switches), or even in the absence of defects due to the inherent structural flexibility of a corner-sharing network (as recently posited for related Pr2Zr2O7 in another Nat. Commun. article)? A key contribution of this manuscript is to precisely correlate defect structures with the physics -- but this necessarily entails explicitly testing not just one model, but many, to show what subset can explain the observed data.

4) Related to point 3, there is no actual evidence in the present manuscript that the annealing changes the oxygen stoichiometry. For example, it is well-known in related materials that annealing at ~1100 C can also reduce, by a large amount, the number of extended antiphase domain boundaries, without a change in stoichiometry. Thermogravimetric analysis (or related) would seem to be needed to support the claim that it is a change in oxygen stoichiometry.

5) To absolutely connect the defect structures to the physics, the manuscript really needs to also compare the measured samples on the basis of the proxy used to date in the field: low temperature specific heat, and, more specifically, the T of the transition (Tmax ~ 0.27 K) and its magnitude. The lack of this does limit the potential acceptance of the results by the community.

6) The manuscript is missing many references (from acta materialia to Nat. Commun. to PRX) on pyrochlore and defect physics. The authors should find and add them to the present work.

In short, the data of this manuscript has the potential to push the understanding of the quantum spin ice $Yb_2Ti_2O_7$, but the above technical points need to be addressed. The authors should be lauded for their openness in sharing the experimental and theoretical data upon request, and providing data dois.

We thank the reviewers for their support and for their constructive criticism. Here we provide a point-by-point response to their reports. We have answered all of their queries, sometimes with new experimental results and analysis. We explain how the suggested changes have been implemented in a substantially revised and improved manuscript that we hope now widely meets the publication criteria for your journal.

Reviewers' comments:

Reviewer #1 (Remarks to the Author):

The paper reports the defect structures in oxygen depleted and Yb stuffed samples of $Yb_2Ti_2O_7$ by means of neutron diffuse scattering investigation. The characteristics of the diffuse scattering are compared with the Monte Carlo calculation results to reveal the structural distortions in both cases are associated with isolated O(2) vacancies replacing the nearest-neighbour Ti^{4+} ions either with the charge compensating Ti^{3+} or with the Yb^{3+}, respectively.
The paper then reports the diffuse scattering observed in as-grown (nominally stoichiometric) sample qualitatively resembles that of the oxygen depleted sample. The most of the diffuse scattering, except the phonon diffuse scattering, is removed when the sample was annealed in oxygen. They then conclude that the dominant defects in the nominally stoichiometric samples are O(2) vacancies.
To correlate these defect structures with the magnetic ground state and correlations, unpolarized and polarized neutron magnetic scattering investigations on these samples have been performed. While at 50 mK the magnetic diffuse scattering from the as-grown sample resembles that of the previously reported spin liquid phase with the rods of scattering along [111] directions, in the annealed sample the diffuse scattering disappeared and ferromagnetic ground state below Tc ~ 425mK is realized. However above Tc the annealed sample also shows diffuse magnetic scattering resembling that of the as-grown sample at a comparable temperature. The oxygen depleted sample does not order down to 50 mK and the diffuse magnetic scattering at this base temperature closely resembles that of the as-grown sample.
In the stuffed sample the transition to the ferromagnetic phase is also suppressed, but in contrast to the oxygen depleted sample it only shows an uniform magnetic diffuse scattering pointing to mainly uncorrelated spins.
In summary the paper highlights the important role of low level intrinsic defects in determining the magnetic ground state of the pyrochlore systems being a model geometrically frustrated systems with quantum fluctuations. The introduction of isolated O(2) vacancies are identified as a mechanism to suppress the ferromagnetic ordered ground state, which is realized in the ultra-pure sample of $Yb_2Ti_2O_7$ obtained after annealing in oxygen.
All the experimental results are carefully compared with the theoretical calculations and thoroughly discussed.
The findings presented in the paper provides a credible scenario in understanding the long standing controversies on the different ground states reported in nominally stoichiometric $Yb_2Ti_2O_7$ samples, hence it can be regarded as a very important contribution to the investigation of pyrochlore compounds as model systems for geometrical frustration in three dimensions. The paper thus definitely warrant publication in Nature Communications, after the authors have considered following points.

1) The presentation of the results in contour diagrams is appropriate to show the global character of the diffuse scattering, but makes the quantitative comparison between the different samples difficult, when the color scales are not given explicitly.
For example
the comparison of Fig.1 (c) and Fig. 2 (a) as described in the main text (on page 2, line 5 from the bottom) ' …, but much weaker.' or
the comparison of Fig. 3 (a) and (b) as described in the main text (on page 3, line 12 from the top) ' …and the diffuse magnetic scattering completely disappeared.'
can not be easily endorsed by just looking at the different contour plots.
A comparison in the form of characteristic cuts using the same scale, as has been employed in the Fig 2 (a) of Ref. 19, could clarify the quantitative differences of the sample dependent diffuse scattering as mentioned in the main text.

All of the 2D plots now have scale bars.

Structural diffuse scattering

The structural diffuse scattering from the stuffed sample (Fig. 1(d)) is stronger than the oxygen-depleted sample (Fig. 1(c)). This can easily be understood since replacing Ti^{4+} ions by Yb^{3+} ions uniformly through the sample results in defect clusters throughout the bulk of the sample. In the case of reduction by hydrogen we find a lower concentration of defects away from the surface (from the colour) and this gives a lower average defect concentration and weaker scattering.

The 2D figures for the as-grown (Fig. 2(a)) and oxygen-annealed (Fig. 2(b)) samples are plotted on the same scale. As the referee suggests, we now include a 1D cut through the data for the oxygen-depleted, as-grown and oxygen-annealed samples as Fig. 2(c).

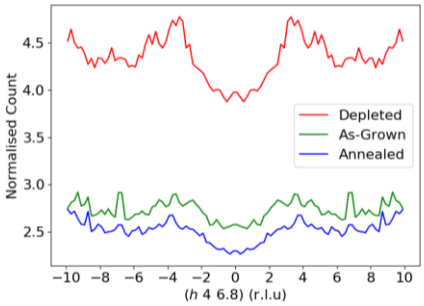

The diffuse scattering from the as-grown sample is clearly much weaker than for the depleted sample. The diffuse scattering from the annealed sample is weaker still, but is not zero, because we detect inelastic scattering from phonons. The inelastic scattering intensity depends upon the scattering geometry, and is expected to differ for each sample. This cut was chosen to minimise inelastic scattering. The broad structural diffuse features at $h \sim \pm 4$ are clearly present for the oxygen-depleted and as-grown samples, but are greatly reduced for the oxygen-annealed sample.

In fact, the best comparison between the as-grown and annealed samples is presented in the 2D plots in Fig. 2(a) and (b) which are on the same scale. The diffuse features near (337) are particularly instructive. This scattering includes relatively sharp features with the symmetry of the underlying structure. This is because they arise from accidental sampling of the phonon

dispersion followed by symmetrisation of the data. In order to tie down this behaviour it is best to look at a single area detector in a single orientation and to compare with a first-principles simulation of the phonon scattering (Fig. S1(b)). In fact, if you look carefully at the corresponding data set for oxygen-annealed $Y_2Ti_2O_7$ in Fig. 2(c) from Sala *et al. Nat. Mater.* **13**, 488-493 (2014) there is also a small inelastic feature from the strong acoustic phonons emerging from (337).

Magnetic diffuse scattering

We also include 1D cuts through the magnetic scattering in Figs 3(a) and (b) in new panels in Figs. 3(c) and (d).

Fig. 3(c). As-grown

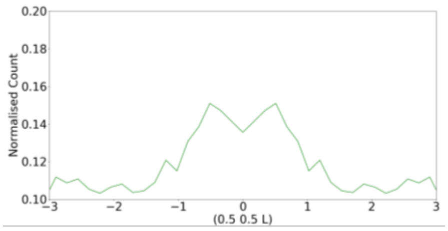

Fig. 3(d). Annealed

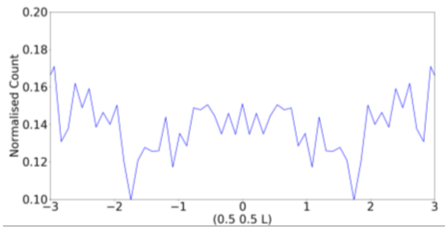

The diffuse magnetic scattering from $-1 < L < 1$ observed for the as-grown sample completely disappeared for the oxygen-annealed sample. Figure 3(a) and (b) are plotted with different scale bars because of the higher background level in Fig. 3(d) from the larger volume of copper in the beam. The dips in the scattering at $L = \pm 1.8$ in Fig. 3(d) result from the grazing-incidence / grazing-exit absorption by the copper plate, see Fig. S2(c).

In addition the information on the size of the samples used for the neutron scattering experiments on Yb2Ti2O4 would be desirable to judge the intensity comparison of the different samples.
For example comparing the contour diagrams of Fig. 4 (a), (c) and (d), the overall spin-flip intensities of the stuffed sample (d) seem to be much higher than in other two samples, if the common color scale is assumed for these diagrams. Does this indicate a larger magnetic moment in the stuffed sample or is it due to the different size of the samples?

Providing an overall scaling of the observed intensities, e.g. on a nuclear Bragg peak, would certainly solve all the problems and would add essential information on the intensity levels of the diffuse structural and magnetic scattering discussed in this paper.

All of the 2D plots have scale bars. The experimental data sets from different crystals have been normalised by crystal size (checking nuclear Bragg peaks) after background subtraction to allow comparison between samples. All calculations include an arbitrary scale factor.

We have replotted Figs. 4(c) and (d) on the same scale as (a). In this way it is possible to compare the intensity of the scattering in each case. In the case of the oxygen-depleted sample, it is clear that the scattering resembles that of the as-grown sample, but is weaker. The weak scattering from the stuffed sample is more washed out and the results do not suggest an increase in the moment size of the Yb^{3+}.

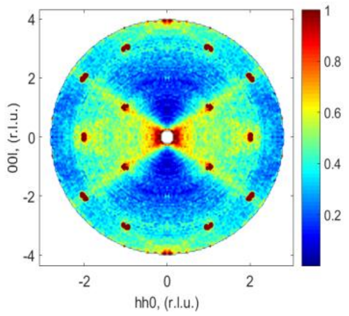

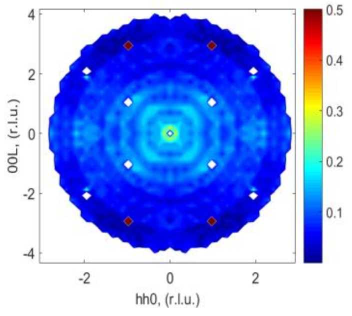

2) Additional refinement results for the as-grown sample in Table 1 may provide important information on the level of O(2) vacancies in ‘ so called’ nominally stoichiometric Yb2Ti2O7 samples.

We have updated Table 1 to include the as-grown sample. Unfortunately, we do not have the sensitivity using single-crystal x-ray diffraction to distinguish between the as-grown and annealed samples. This was also the case for $Y_2Ti_2O_7$ [Sala *et al. Nat. Mater.* **13**, 488-493 (2014)]. We need structural diffuse scattering to determine the defect structures.

The following is the updated version of Table 1.

	Stuffed	Depleted	As-grown	Annealed
Colour	Yellow/Brown	Black	Brown	Transparent
Space group	Fd-3m	Fd-3m	Fd-3m	Fd-3m
Lattice parameter	10.0838(14)	10.0216(16)	10.0271(2)	10.0313(17)
Yb	1	1	1	1
Yb2	0.26(3)	0	0	0
Ti	0.74(3)	1	1	1
O(1)	1	1	1	1
O(2)	0.96	0.96(3)	1	1
x	0.3397(14)	0.3300(6)	0.3308(2)	0.3309(10)
R	4.01	1.92	4.23	2.60
R_W	4.90	2.60	4.87	2.78

3) Are the error bars for Fig.3 (c) smaller than the data points?

The error bars for the temperature dependence of the integrated intensities of the (113) Bragg reflection normalised to unity at *T* ~ 1K have been added to Fig. 3(c), which is now Fig. 3(e). The error bars for the flipping ratios are smaller than the data points. [The flipping ratios are now taken from the same data sets as the peak intensities and, because they have had longer for the temperature to stabilise, they have less scatter than before.] We have replaced Fig. 3(c) by this version, now Fig. 3(e).

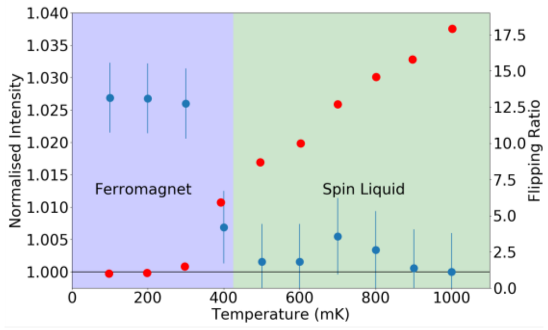

Likewise, are the error bars in Fig 3 (d) for the (002) and (220) intensities smaller then the data points?

The error bars for (002) and (220) are smaller than the data points. For (111), (113) and (222) the magnetic intensity is on top of much larger structural scattering and, therefore, subtraction of two large numbers gives larger error bars. This is now Fig. 3(f).

4) Typo: on page 2, line 19 from the top, ' ….. presented in Fig. 1(b) ….' should read ' ….. presented in Fig. 1(c) ….' .

Many thanks for pointing out this typo, which is now corrected in the revised manuscript.

Reviewer #2 (Remarks to the Author):

This manuscript describes a study of the influence of structural and chemical disorder on the spin correlations of Yb2Ti2O7. Yb2Ti2O7 is a material which has attracted a lot of interest over an extended period due to its magnetic frustration and possible link to quantum spin liquid phases. Sample dependence has been a significant issue in studies of Yb2Ti2O7 and the role of disorder in determining its magnetic properties is not well understood.

This work adds new evidence to the discussion of disorder in Yb2Ti2O7 by presenting measurements of both the structural and magnetic diffuse scattering, for various Yb2Ti2O7 samples and showing the relationship between the two. In particular, it is shown that two different types of disorder (oxygen deficiency and Yb stuffing) have different effects. Both types of disorder suppress the ferromagnetic ordered ground state but while oxygen deficiency promotes a correlated spin liquid state, Yb stuffing suppresses correlations altogether. The authors' present evidence that the true ground state of stoichiometric Yb2Ti2O7 is ferromagnetically ordered.

This work provides a lot of new evidence about the role of disorder in Yb2Ti2O7 and its magnetic ground state and it will be of considerable interest to the community studying this material. Since Yb2Ti2O7 has become something of an important test case for frustrated quantum magnets in 3 dimensions, these results will also be of broader interest. More generally, the interplay of frustration, quantum fluctuations and quenched disorder is an increasingly studied topic at present and this work is of interest from that point of view. I therefore think that this work is of sufficient novelty and importance to merit publication in Nature Communications, if the authors can satisfactorily address the points I have listed below.

There are a few things which I think the authors need to address. These are:

1) The authors estimate the transition temperature for their oxygen annealed sample at 425mK. This is much higher than previous estimates of Tc for Yb2Ti2O7 which generally fall between 200-300mK. Most of the published estimates of Tc are based on a sharp peak in the heat capacity whereas the present paper uses the onset of a magnetic Bragg peak. It is therefore important to know whether the difference in estimates of Tc is due to the different method of estimation or whether the authors' sample really has a much higher Tc than everyone else's. This would be clarified if the authors could show some heat capacity measurements for their samples for comparison with the existing literature.

The heat capacity for a single crystal of $Yb_2Ti_2O_7$ cut from the same boule and annealed in oxygen under identical conditions has already been published in Fig. 2 and Supplementary Fig. S19 of Thompson *et al*. *Phys. Rev. Lett.* **119**, 057203 (2017). This sample exhibits a sharp peak at T_C ~ 214 mK. Hence our sample has a T_C in the range 200 – 300 mK in common with the best single crystals in the literature, e.g. Chang *et al*. *Nat. Commun.* **3** 992 (2012) and Arpino, K. E. *et al*. *Phys. Rev. B* **95**, 094407 (2017).

Thompson, J. D. *et al. Phys. Rev. Lett.* **119**, 057203 (2017)

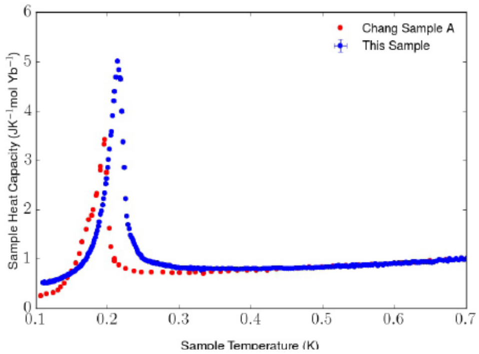

The onset temperature quoted in the manuscript, T_C ~ 425 mK was estimated using the order parameter in Fig. 3(e) upon heating. In a neutron diffraction measurement from a large single crystal connected to a copper base only by copper wires it is challenging to achieve equilibrium at a few hundred mK. Furthermore, the transition to the ferromagnetic phase is first order and there is very strong hysteresis. This phenomenon has been investigated previously by Yasui *et al*. (2003) and Gaudet *et al* (2016). The neutron measurements display very strong hysteresis and, similarly to our results, the disappearance of the ferromagnetic Bragg intensity is at T ~ 400 mK while the peak in the susceptibility or heat capacity is between 200 – 300 mK. Furthermore, in a neutron measurement without energy analysis it is possible to overestimate the transition temperature compared to static probes if the

measurement integrates over both static and low energy fluctuations. Hence the difference in T_Cs are due to the method of measurement.

Yasui, Y. *et al. J. Phys. Soc. Jpn.* **72**, 3014 (2003)

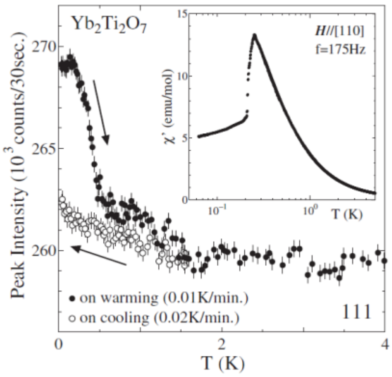

Gaudet, J. *et al. Phys. Rev. B* **93**, 064406 (2016)

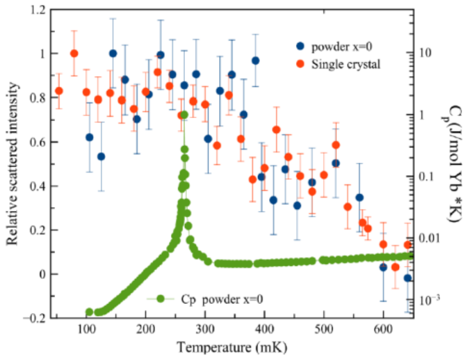

We have added the following clarification to the manuscript

"We note that a single crystal of $Yb_2Ti_2O_7$ cut from the same boule and annealed in oxygen under identical conditions exhibits a sharp peak in its heat capacity at T_C ~ 214 mK[13]. This is consistent with the purest samples in the literature[2,19]. The different T_C from neutron diffraction can readily be explained by either the large hysteresis observed previously for this transition[6,9] or possibly the accidental inclusion of low energy fluctuations."

2) The authors' neutron scattering refinement finds a splayed ferromagnetic order with the splaying being of an "all in all out" (AIAO) rather than "two in two out" (2I2O) form. They state that this AIAO splayed FM order is reproduced in their classical Monte Carlo simulations. This surprises me. The classical phase diagram of the pyrochlore nearest neighbor anisotropic exchange model has been studied in some detail in previous works (particularly Ref. 31 of the manuscript) with only one splayed FM phase found, and this phase has 2I2O splaying. In fact, 2I2O rather than AIAO splaying is expected on quite general symmetry grounds. I also note that Ref. 26 which did Monte Carlo simulations using the same parameter set as the present work seems to find 2I2O splaying (see Figs. 7 c and d of Ref. 26). Given all this, it is quite a striking result if the authors have an FM phase with AIAO splaying in their classical simulations. They should present further evidence to support this and,
ideally, an explanation of how such a state can be stabilised. For supporting evidence, I think it would be useful if they showed a plot from their simulations of the sum of the spin projections on their local <111> axes (spin ice axes) which should vanish in a 2I2O splayed state but be finite in an AIAO splayed state.

We thank the referee for spotting a problem in the way we explained our results in the manuscript, and we take the opportunity to clarify the matter. We have replaced the following sentence in the manuscript

"For this model below T_C, the system orders in the all-in all-out splayed ferromagnetic phase, in agreement with our experiment."

with

“For this model below T_C the system orders in a ferromagnetic phase in agreement with previous MC simulations[30,34-35]. These exchange parameters place the system close to the boundary with the antiferromagnetic ψ_3 phase, which has vanishing (002) scattering and strong (220) intensity, in agreement with our experiment.”

The ground state of the Monte Carlo is indeed the splayed ferromagnet studied in Refs. [30] and [35].

However, the best parameters identified by Robert *et al*. are close to the phase boundary between the FM and the ψ_3 phase. (Notice that in Ref. [30] their optimal J_2 = -0.32 and Fig.7 are slightly at odds with the behaviour described in Sec. III D of their manuscript, and our Monte Carlo agrees with the latter).

As we get closer and closer to the FM/ψ_3 phase boundary, the system exhibits stronger and stronger antiferromagnetic correlations (witnessed by a growing (220) scattering intensity with lack of (002) scattering, as shown for instance in the panels (e)-(f)-(g) of Fig. 24 in Ref. [35]. Please note that in Figs. 20-22 in Ref. [35] the [00*l*] and [*hh*0] axes are accidentally swapped, bringing these figures into agreement with our results, see

H. Yan, O. Benton, L. Jaubert, N. Shannon *Phys. Rev. B* **97**, 219905(E) (2018)

Erratum: Theory of multiple-phase competition in pyrochlore magnets with anisotropic exchange with application to $Yb_2Ti_2O_7$, $Er_2Ti_2O_7$, and $Er_2Sn_2O_7$ [*Phys. Rev. B* **95**, 094422 (2017)]

These are the "all-in, all-out" correlations that we observe in our experiments and Monte Carlo, superposed to an overall FM order.

We apologise to the referee about our confusing statement that appeared to attribute the all-in all-out correlations to the FM phase directly. We hope that the changes to the manuscript have now made our discussion sufficiently clear.

3) When the authors write in the introduction "...the latest values of the g-tensor show that the moments are more easy plane than easy axis", I think this slightly misrepresents the discussion about Yb2Ti2O7 in the literature. It has been known for a long time that the g-tensor is predominantly easy plane (see e.g. Hodges et al, JPCM 13, 9301 (2001)). While estimates of the g-tensor parameters have varied a bit, every study of which I am aware has found g_{xy}>g_z. The point of significant disagreement has not been the g-tensor but the relative strength of different exchange interactions with Ref. 1 of the manuscript finding quite a strong Ising term, but Refs. 13 and 26 finding that the Ising term is rather weak. I think the introduction should be modified to better reflect the discussion in the literature.

We thank the referee for pointing out an ambiguity in our sentence – in referring to the latest values it could be implied that the situation has recently changed, whereas the fact that the moments are more easy plane than easy axis has been apparent for some time. We are happy to modify this sentence in the manuscript and refer to the original publication.

Hence, we have replaced

“the latest values of the *g*-tensor show that the moments are more easy-plane than easy-axis[4]”

by

“the values of the anisotropic *g*-tensor show that the moments are more easy-plane than easy-axis [Hodges *et al*. *J. Phys.: Condens. Matter* **13**, 9301 (2001)]”

4) The abstract describes Yb2Ti2O7 as the "quantum analogue" of a classical spin ice. I am not sure this statement is justified. Although connections have been made between Yb2Ti2O7 and spin ice in the past, if recent estimates of the exchange parameters are correct (e.g. Ref. 13 of the manuscript) then the spin-ice-like Ising exchange in Yb2Ti2O7 is quite weak, which would suggest that the physics of Yb2Ti2O7 has little to do with spin ice, apart from being on the same lattice.

We have removed from the abstract the sentence that suggested $Yb_2Ti_2O_7$ to be a quantum analogue of classical spin ice.

Since this paper provides new and interesting insights into a topic of quite broad interest, I will be happy to recommend it for publication in Nature Communications if the authors can satisfactorily address the points above.

Reviewer #3 (Remarks to the Author):

The rare earth pyrochlore magnets are benchmark materials for exploring the physics of geometrically frustrated lattices, and the interplay between single ion and emergent collective magnetic states. Recently, much work on compounds including Nd2Zr2O7, Pr2Zr2O7, and Yb2Ti2O7 (in journals from Nat. Commun. to PRX and PRL and even Acta Materialia) has shown that seemingly small variations in synthetic procedure and composition have an outsize impact on the resultant physics. As such, this is a timely contribution.

This manuscript's major claims are:

1) that defects play a pivotal role in determining the magnetic ground state of the putative quantum spin ice Yb2Ti2O7,
2) that the ferromagnetic state is the "true" ground state,
3) that oxygen vacancies can exist and favor the formation of a spin liquid (SL) state,
4) and that Yb stuffing suppresses the SL state.

Claim 1 has been known for many years, particularly with respect to the divergence (or not) in specific heat at T ~ 0.27 K, and claim 2 appears to be the community consensus. It is on the third and fourth claims that this manuscript aims to move the field forward -- prior work on Yb2Ti2O7 has found numerous types of defect structures, but the direct correlation between these and the physics has remained elusive. Part of the challenge is simply the sheer number of observed defect structures -- not just point defects (e.g. site mixing, oxygen

vacancies) but also more recently a range of extended defects (antiphase domain boundaries observed in STEM, cooperative tetrahedral motion observed in electron diffraction).

Diffuse scattering is an excellent tool to probe both point and extended defect structures, and the data in this manuscript appears comprehensive. Further, neutron scattering (particularly the spin-flip variety) is a superb tool by which to probe the magnetic state of Yb2Ti2O7. Thus in principle the *data* of this manuscript provide the pieces needed resolve a number of long-standing questions about this material.

However, as it stands, the present level of analysis of the data, as well as a couple of defining measurements and references to the literature, are missing, and significantly weaken this manuscript. Specific technical points in no particular order:

1) For the structures reported in Table 1 from X-ray data: is this from powder or single crystal data? in either case, significant information about how the data was collected, reduced, and analyzed, is missing. If it is single crystal data, how the integration and scaling was done, how absorption corrections were applied, and how symmeterization was done is all missing; as are the unweighted R and chi^2 metrics, etc. Whether powder of single crystal, the refinement also includes atomic displacement parameters which are missing and not reported (and correlate with the sought after occupancies!), missing uncertainties in the O(2) occupancies, and missing atomic coordinates for each crystallographically distinct atomic position (including the critical refinable oxygen position).

Further details of the single-crystal measurements and refinements have been added to the Methods section and the CIF files are now included in the Supplementary Information:

"The average structures were determined by single-crystal x-ray diffraction using a Molybdenum source Oxford Diffraction diffractometer at Royal Holloway. A large CCD detector captures full reciprocal space coverage to a real space resolution of 0.6Å. 3D profile analysis of each reflection is performed using the CrysAlisPro software [Agilent (2014)] and refinements of the average structure, including anisotropic thermal parameters, are performed using the Jana2006 software [Petricek *Z. Kristallogr.* **229**, 345-352 (2014)]. All refinements had R-factors less than 5%, exhibiting excellent fits to the data. Full details are available in the CIF files included in the Supplementary Information."

We agree that it is difficult to determine occupancies in a robust manner. In the case of oxygen, we are not able to obtain reliable occupancies from Bragg diffraction data alone, or to distinguish between O(1) and O(2) vacancies. That is one reason why in addition diffuse scattering is required.

The O(2) position in our refinements for the stoichiometric sample $x = 0.3309(10)$ agrees with our geometry optimisation for $Yb_2Ti_2O_7$ using first-principles density-functional theory, and with previous refinements using powder neutron diffraction [Ross *Phys. Rev. B* **86**, 174424 (2012)] and powder synchrotron x-ray diffraction [Baroudi *Phys. Rev. B* **92**, 024110 (2015)].

We have included an updated version of Table 1, with further details on atomic coordinates and new data on the as-grown sample:

	Stuffed	Depleted	As-grown	Annealed
Colour	Yellow/Brown	Black	Brown	Transparent
Space group	Fd-3m	Fd-3m	Fd-3m	Fd-3m
Lattice parameter	10.0838(14)	10.0216(16)	10.0271(2)	10.0313(17)
Yb	1	1	1	1
Yb2	0.26(3)	0	0	0
Ti	0.74(3)	1	1	1
O(1)	1	1	1	1
O(2)	0.96	0.96(3)	1	1
x	0.3397(14)	0.3300(6)	0.3308(2)	0.3309(10)
R	4.01	1.92	4.23	2.60
R_W	4.90	2.60	4.87	2.78

Table 1 | Refinement of the average structures of stuffed $Yb_2(Ti_{2-x}Yb_x)O_{7-x/2}$, oxygen-depleted $Yb_2Ti_2O_{7-\delta}$, as-grown and oxygen-annealed $Yb_2Ti_2O_7$ from the single-crystal X-ray structure factors measured at T ~ 300 K. The fractional coordinates are given within the second origin choice of the Fd-3m space group: Yb1 (0.5,0.5,0.5), Yb2 (0,0,0), Ti (0,0,0), O(1) (0.375,0.375,0.375), O(2) (*x*,0.125,0.125). The goodness of fit is described by the crystallographic *R*-factor and the weighted *R*-factor, R_W.

2) Also, this is a cubic material so a single measurement of the lattice parameter 100% correlates with various common diffractometer alignment errors -- how was this accounted for in mesauring the cubic parameters? What was actually used for the X-ray measurements "The average structures were determined using x-ray diffraction at Royal Holloway" is not an adequate description of what was used and how the measurements were carried out.

We did not use a single measurement using a point detector to determine the lattice parameters. Our single-crystal diffractometer measures large numbers of reflections at many orientations and is calibrated against a standard sample and, therefore, we have no reason to expect large systematic errors.

We have provided more details on the measurements in the Methods section, see point 1) above.

3) The agreement between the models and the data appear reasonably good in Fig. 1.

Crucially, however, this does not mean that other physically plausible models can be ruled out. More specifically, it appears a majority of the diffuse scattering appears to arise due to correlated movement of metal ions. This might be due to a point defect (e.g. an oxygen vacancy or a Yb-for-Ti stuffing, as claimed), but could it not also arise around the other types of defects observed in the literature -- e.g. antiphase domain boundaries (where definition of A and B sites switches), or even in the absence of defects due to the inherent structural flexibility of a corner-sharing network (as recently posited for related Pr2Zr2O7 in another Nat. Commun. article)? A key contribution of this manuscript is to precisely correlate defect structures with the physics -- but this necessarily entails explicitly testing not just one model, but many, to show what subset can explain the observed data.

The techniques of traditional diffraction and diffuse scattering both refine structural models rather than provide unique solutions. The best one can do is to obtain a good fit with physically reasonable parameters, and to not find a better model. There is always the possibility that another ingenious model fits the data at least as well. We can, however, rule out the two other types of defect mentioned here.

The presence of antiphase domain boundaries leads to sharp Bragg peaks at reflections with even *h*, *k*, *l* corresponding to the average fluorite lattice, and diffuse scattering at the remaining pyrochlore reflections [Lau *et al*. *Phys. Rev. B* **76**, 054430 (2007)]. We can rule this out since all of our samples exhibit sharp pyrochlore reflections corresponding to long-range ordering of A and B sites. Furthermore, we do not find any superlattice reflections.

Static disorder due to the flexibility of the corner-sharing network described in Trump *et al*. *Nat. Commun.* 9:2619 (2018) can also be ruled out in our case. Our oxygen-annealed, nominally stoichiometric sample only exhibits dynamic disorder, since the diffuse scattering can be accounted for by phonon scattering. It is interesting to note that the static disorder in Trump *et al*. is based upon the dynamic disorder in β-cristobalite, which undergoes a structural phase transition to static α-crystobalite [Hatch *et al*. *Phys. Chem. Minerals* **17**, 554 (1991)]. No such structural phase transition is observed in pyrochlores, presumably because the kagome layers are filled. Our first-principles density-functional calculations for $Yb_2Ti_2O_7$ demonstrate that the pyrochlore structure is stable and there are no structural distortions in the stoichiometric system.

We added the following clarification to the Methods:

"We were not able to reproduce the observed diffuse scattering using simulations with other point defects, such as O(1) vacancies[22]. We were further able to rule out antiphase domain boundaries[44], since all of the pyrochlore Bragg reflections were sharp, and static disorder from the flexibility of the corner-sharing network[45], since structural diffuse scattering is absent in our stoichiometric sample."

In this paper we have deliberately grown samples with oxygen deficiency and stuffing, and this is helpful in tying down the defect structure.

In the case of oxygen deficiency, one might expect vacancies on either O(1) or O(2) sites, and we have tested these models exhaustively for different displacements of surrounding ions. We were not able to achieve agreement with the data for O(1) vacancies, and the absence of an increase in lattice parameter seems to rule that model out. In the case of O(2) vacancies, we tried a large number of displacements of surrounding ions (so-called "size effects") and

we noted in the manuscript that the resulting Ti^{3+}-O(2) bond length, 2.03Å, agrees with the values in the literature [Abrahams *Phys. Rev.* **130**, 2230 (1963)]. We also noted in the manuscript that a powder neutron diffraction study of $Yb_2Ti_2O_7$ reduced at low temperature in a topotactic reaction with CaH_2 also finds O(2) vacancies [Blundred *et al*. *Angew. Chem.* **43**, 3562 (2004)].

In the case of stuffing, the replacement of Ti^{4+} by Yb^{3+} ions results in oxygen vacancies, and we concluded that oxygen ions were removed from adjacent O(2) sites rather than O(1) sites. Subsequent to our analysis we have become aware of a study of $Yb_2(Ti_{2-x}Yb_x)O_{7-x/2}$ using first–principles density-functional theory [Ghosh *et al*., *Phys. Rev. B* **97**, 245117 (2018)] which concluded that stuffing is accompanied by the formation of oxygen vacancies, and that vacancies on O(2) sites are energetically stable in comparison to vacancies on O(1) sites (lower in energy by 0.4eV per Yb tetrahedron!) in agreement with our conclusion. We now include the following sentence in the manuscript:

"Recently, calculations for $Yb_2(Ti_{2-x}Yb_x)O_{7-x/2}$ using density-functional theory have confirmed that O(2) vacancies are energetically stable relative to O(1) vacancies [Ghosh *et al*., *Phys. Rev. B* **97**, 245117 (2018)]."

Our models certainly have the virtue of simplicity and, given the relatively small number of adjustable parameters, the agreement with the data is quite remarkable.

4) Related to point 3, there is no actual evidence in the present manuscript that the annealing changes the oxygen stoichiometry. For example, it is well-known in related materials that annealing at ~1100 C can also reduce, by a large amount, the number of extended antiphase domain boundaries, without a change in stoichiometry. Thermogravimetric analysis (or related) would seem to be needed to support the claim that it is a change in oxygen stoichiometry.

We have performed further measurements and added the following clarification to the Methods section:

"Thermogravimetric analysis reveals a change in the oxygen stoichiometry of 0.38±0.14%."

5) To absolutely connect the defect structures to the physics, the manuscript really needs to also compare the measured samples on the basis of the proxy used to date in the field: low temperature specific heat, and, more specifically, the T of the transition (Tmax ~ 0.27 K) and its magnitude. The lack of this does limit the potential acceptance of the results by the community.

The heat capacity for a single crystal of $Yb_2Ti_2O_7$ cut from the same boule and annealed in oxygen under identical conditions has already been published in Fig. 2 and Supplementary Fig. S19 of Thompson *et al*. *Phys. Rev. Lett.* **119**, 057203 (2017). This sample exhibits a sharp peak at T_C ~ 214 mK. Hence our sample has a peak in its heat capacity at comparable temperature, of similar width and magnitude to the best single crystals in the literature, e.g. Chang *et al*. *Nat. Commun.* **3** 992 (2012) and Arpino, K. E. *et al*. *Phys. Rev. B* **95**, 094407 (2017).

Thompson, J. D. *et al. Phys. Rev. Lett.* **119**, 057203 (2017)

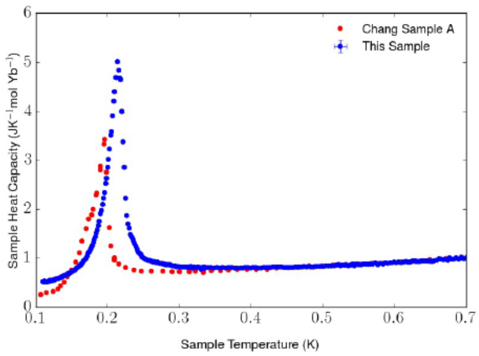

We have added the following clarification to the manuscript:

"We note that a single crystal of $Yb_2Ti_2O_7$ cut from the same boule and annealed in oxygen under identical conditions exhibits a sharp peak in its heat capacity at T_C ~ 214 mK[13]. This is consistent with the purest samples in the literature[2,19]. The different T_C from neutron diffraction can readily be explained by the large hysteresis observed previously for this transition[6,9] or possibly the accidental inclusion of low energy fluctuations."

6) The manuscript is missing many references (from acta materialia to Nat. Commun. to PRX) on pyrochlore and defect physics. The authors should find and add them to the present work.

We have added the following references:

Trump *et al. Nat. Commun.* 9:2619 (2018)

Lau *et al. Phys. Rev. B* **76**, 054430 (2007)

Baroudi *et al. Phys. Rev. B* **92**, 024110 (2015)

Ghosh *et al. Phys. Rev. B* **97**, 245117 (2018)

Peçanha-Antonio *et al. Phys. Rev. B* **96**, 214415 (2017).

Andreano *et al. Phys. Rev. B* **81**, 014406 (2010)

Sen *et al. Phys. Rev. Lett.* **114**, 247207 (2015)

Wen *et al. Phys. Rev. Lett.* **118**, 107206 (2017)

Savary *et al. Phys. Rev. Lett.* **118**, 087203 (2017)

Martin *et al. Phys. Rev. X* **7**, 041028 (2017)

Chern *et al.* arXiv:1806.01276

In short, the data of this manuscript has the potential to push the understanding of the quantum spin ice Yb2Ti2O7, but the above technical points need to be addressed. The authors should be lauded for their openness in sharing the experimental and theoretical data upon request, and providing data dois.

We thank all three referees for their helpful comments.

REVIEWERS' COMMENTS:

Reviewer #1 (Remarks to the Author):

The authors have provided a comprehensive point-by-point response to referees' reports and revised the manuscript accordingly including additional results and analysis.
The revised manuscript thus definitely warrants publication in Nature Communications.

Reviewer #2 (Remarks to the Author):

Having read the revised manuscript and the authors' response to the first round of reports, I find the authors' response satisfactory. I believe they have clarified all of the issues raised in my report and in the reports of the other reviewers.

There is one point on which I would like to press the authors for further clarification:

After their response to my previous report, the interpretation of the authors' simulation results seems clear. They find a ferromagnetic order with "2I2O" splaying, but the exchange parameters are close to a phase boundary with an antiferromagnetic phase. The authors seem to suggest that this proximate antiferromagnetic phase could be responsible for the intensity at 220 and the lack of intensity at 200 which led the authors to rule out a 2I2O splayed ferromagnet from their refinement of the magnetic Bragg peaks [Fig. 3f and associated discussion in the text].

So what are the authors saying about the ground state of Yb2Ti2O7? Are they saying that it is a 2I2O ferromagnet and that the fluctuations from the proximity of a neighbouring phase make it appear to have the wrong distribution of Bragg peak intensities? Or are they saying that there are actually regions of the sample which are ordered in the antiferromagnetic phase? Or something else?

Although I would like the authors to clarify this point, it is not crucial to the main purpose of their paper, which is about the effects of disorder. I therefore will not insist on any further revision.

This is a well written paper adding important new evidence to the discussion of an interesting topic, and since the authors have generally given convincing responses to the last round of reports, I recommend that this paper be published in Nature Communications.

Reviewer #3 (Remarks to the Author):

In this revision, the authors have comprehensively addressed the reviewer critiques, adding new data where appropriate, and enhancing the quantitative nature of the data presented when needed. It is now in a form that will significantly push the field forward, and is highly suited for publication in Nat. Comm.

REVIEWERS' COMMENTS:

Reviewer #1 (Remarks to the Author):

The authors have provided a comprehensive point-by-point response to referees' reports and revised the manuscript accordingly including additional results and analysis.
The revised manuscript thus definitely warrants publication in Nature Communications.

Reviewer #2 (Remarks to the Author):

Having read the revised manuscript and the authors' response to the first round of reports, I find the authors' response satisfactory. I believe they have clarified all of the issues raised in my report and in the reports of the other reviewers.

There is one point on which I would like to press the authors for further clarification:

After their response to my previous report, the interpretation of the authors' simulation results seems clear. They find a ferromagnetic order with "2I2O" splaying, but the exchange parameters are close to a phase boundary with an antiferromagnetic phase. The authors seem to suggest that this proximate antiferromagnetic phase could be responsible for the intensity at 220 and the lack of intensity at 200 which led the authors to rule out a 2I2O splayed ferromagnet from their refinement of the magnetic Bragg peaks [Fig. 3f and associated discussion in the text].

So what are the authors saying about the ground state of Yb2Ti2O7? Are they saying that it is a 2I2O ferromagnet and that the fluctuations from the proximity of a neighbouring phase make it appear to have the wrong distribution of Bragg peak intensities? Or are they saying that there are actually regions of the sample which are ordered in the antiferromagnetic phase? Or something else?

Although I would like the authors to clarify this point, it is not crucial to the main purpose of their paper, which is about the effects of disorder. I therefore will not insist on any further revision.

As stated in the current version of the manuscript, we have no evidence for 2I2O correlations. Our results are instead consistent with either the AIAO splayed FM phase, or the collinear FM coexisting with the ψ_3 AF phase. We have corrected a typographic mistake "collinear splayed ferromagnet" to "collinear ferromagnet" which may have led to some confusion.

This is a well written paper adding important new evidence to the discussion of an interesting topic, and since the authors have generally given convincing responses to the last round of reports, I recommend that this paper be published in Nature Communications.

Reviewer #3 (Remarks to the Author):

In this revision, the authors have comprehensively addressed the reviewer critiques, adding new data where appropriate, and enhancing the quantitative nature of the data presented when needed. It is now in a form that will significantly push the field forward, and is highly suited for publication in Nat. Comm.

We thank all three reviewers for their supportive comments.
